# Transcriptomics, lipidomics, and single-nucleus RNA sequencing integration: exploring sphingolipids in MASH-HCC progression

**DOI:** 10.1186/s13578-025-01362-5

**Published:** 2025-03-08

**Authors:** Jing Zeng, Grayson Way, Nan Wu, Xixian Jiang, Yun-ling Tai, Derrick Zhao, Lianyong Su, Qianhua Yan, Xuan Wang, Emily C. Gurley, Phillip B. Hylemon, Sayed Obaidullah Aseem, Arun J. Sanyal, Jiangao Fan, Huiping Zhou

**Affiliations:** 1 Department of Microbiology & Immunology, Virginia Commonwealth University, Richmond Veterans Affairs Medical Center, 1220 East Broad Street, Richmond, VA MMRB-5044, 23298-0678 USA; 2 Department of Gastroenterology, Xinhua Hospital, Shanghai Jiao Tong University School of Medicine, Shanghai, 200092 China; 3 Stravitz-Sanyal Institute for Liver Disease & Metabolic Health, School of Medicine, Virginia Commonwealth University, Richmond, VA USA; 4 Division of Gastroenterology, Hepatology and Nutrition, Department of Internal Medicine, Medical College of Virginia, Virginia Commonwealth University, Richmond, VA USA

**Keywords:** Transcriptomics, Lipidomics, Single-nucleus RNAseq, Nanostring gene profiling, Sphingolipids, MASLD, MASH, HCC

## Abstract

**Background & aims:**

Metabolic dysfunction-associated steatotic liver disease (MASLD) encompasses various conditions, ranging from simple steatosis to metabolic dysfunction-associated steatohepatitis (MASH) and cirrhosis. MASLD is a significant risk factor for hepatocellular carcinoma (HCC) and is rapidly becoming the primary cause of liver transplantation. Dysregulated sphingolipid metabolism has been linked to the development of MASH-HCC. However, detailed insight into the sphingolipid profiles and cell type-specific changes in key genes involved in sphingolipid metabolism remains limited and forms the primary focus of this study.

**Approaches & results:**

This study used the well-characterized diet-induced MASH-HCC mouse model (DIAMOND). Total RNA sequencing data, NanoString nCounter^®^ Gene profiling, and single-nucleus RNA sequencing (snRNA-seq) GEO data (GSE225381) were used in characterizing gene regulation in MASH-HCC progression. Sphingolipids in the serum and liver were profiled using targeted lipidomics. RNA data analysis showed dysregulation of key genes involved in sphingolipid metabolism, including ceramide synthase 6 (Cers6), serine palmitoyltransferase long chain base subunit 2 (Sptlc2), sphingosine kinase 2 (SphK2), and sphingosine-1-phosphate receptor 1–3 (S1pr1-3) which paralleled significant changes in sphingolipid composition and levels in both serum and liver. Furthermore, TCGA-LIHC patient data were analyzed and potential prognostic genes for MASH-HCC were identified using univariate and multivariate Cox analysis. The multivariate Cox analysis underscored the prognostic significance of several genes related to sphingolipid metabolism, including CERS6, SPTLC2, and S1PR1.

**Conclusion:**

Our findings provided valuable insights into the role of sphingolipids in the progression of MASH to HCC. Specific serum and liver sphingolipid profiles may serve as valuable biomarkers for diagnosis and prognosis in MASH-HCC.

**Supplementary Information:**

The online version contains supplementary material available at 10.1186/s13578-025-01362-5.

## Introduction

The global increase in obesity, type 2 diabetes, and metabolic syndrome has led to a marked increase in metabolic dysfunction-associated steatotic liver disease (MASLD) [1]. This rise is accompanied by a growing incidence of liver cancer, particularly hepatocellular carcinoma (HCC), closely associated with MASLD, and metabolic-dysfunction-associated steatohepatitis (MASH) [2]. The progression from MASH to HCC in patients with MASLD is complex and not fully understood. Due to the underdiagnosis and insufficient attention to MASLD, only a limited number of MASLD patients are monitored for the development of HCC. The current guidelines do not suggest routine HCC screening for those with MASLD or MASH unless liver cirrhosis has developed, often leading to a late-stage diagnosis of MASLD-associated HCC with limited treatment options [3–5]. Identifying MASLD patients with a higher HCC risk remains a significant clinical challenge. Implementing lifestyle and pharmacological interventions could potentially prevent HCC in the MASLD population [4, 6]. However, the development of effective drugs specifically targeting MASLD-associated HCC is still in the early stages, with few options currently available [7]. A thorough understanding of the pathophysiological mechanisms and disease phenotypes associated with MASH-HCC is essential for pinpointing precise diagnostic markers and developing effective treatment strategies.

In the past decades, numerous studies have focused on the roles played by specific cell types and individual pathways in MASH-HCC. While these targeted approaches provide detailed insights into specific molecules and pathways, they have also led to varied findings across different studies. However, the advent of advanced deep sequencing technologies, such as total RNA sequencing (RNAseq), single-cell RNAseq (scRNAseq) or single-nucleus RNAseq (snRNAseq), and NanoString’s multiplex direct digital counting, has broadened our capacity to investigate the complex and diverse functions of genes and signaling pathways across various cell types in the progression of MASH-HCC [8, 9]. Given that MASLD is a metabolic disorder, it is plausible that the progression of MASH-HCC is linked with an interactive network among various hepatic cells and their microenvironment rather than from the independent activity of individual cell types [9–11]. Therefore, changes in the population and functionality of specific liver cell types and gene expression patterns may play unique roles in disease progression [12]. Identification of cell-type specific changes in gene expression is crucial for unraveling the complex mechanisms from MASH to HCC.

Sphingolipids, as essential components of cell membranes, play a pivotal role beyond simply maintaining barrier integrity and fluidity. These bioactive lipids are critical regulators in various biological processes (BPs), such as cell proliferation, apoptosis, and senescence. The biosynthetic and metabolic pathways of sphingolipids are well-characterized. Two major bioactive sphingolipids, ceramide and sphingosine-1-phosphate (S1P), have been identified as central signaling molecules regulating various cellular functions [13]. Recent studies have revealed that sphingolipid-mediated signaling pathways are intimately associated with tumor growth and resistance to chemotherapy drugs in different types of cancers [14]. However, the specific role of sphingolipids in the progression of MASLD-HCC is still largely unexplored.

In this study, we utilized the well-characterized diet-induced mouse model for MASLD to identify the key signaling pathways driving the progression of MASH-HCC. By combining total RNA transcriptomics, targeted Lipidomics, NanoString gene profiling, and snRNAseq, we were able to identify cell type-specific changes in sphingolipid metabolism during MASH-HCC progression. Our findings provide novel insights into the role of sphingolipid-mediated signaling pathways in promoting MASH-HCC progression and the potential use of sphingolipid profiles as prognostic markers for MASH-HCC.

## Materials and methods

### Animal experiments

This study utilized DIAMOND mouse models derived from a hybrid strain of C57BL/6J and 129S1/SvlmJ (ages 21 to 24 weeks, both sexes) [15]. All the animal protocols were approved by the Institutional Animal Care and Use Committee of the Richmond VA Medical Center and Virginia Commonwealth University, and the experiments were conducted with the ethical standard for animal research.

### Quantification of sphingolipids in serum and liver tissues

Serum and liver tissues were analyzed for sphingolipid content following methods previously established by our laboratory [16]. The types and concentrations of sphingolipids in these samples were quantified using a Shimadzu LC-MS/MS 8600 system, and data acquisition and processing were carried out using Lab Solutions software [16, 17].

### Statistical analysis

Data are presented as mean ± SEM from at least three independent experiments. Non-parametric Mann-Whitney test was applied for variables that were not normally distributed, whereas normally distributed variables were assessed using an unpaired t-test with GraphPad Prism (version 8; GraphPad Software Inc., San Diego, CA). Additional statistical analyses were performed using R software (version 4.0.3; R Foundation for Statistical Computing, Vienna, Austria). A p-value of less than 0.05 was considered statistically significant.

Additional details on materials and methods can be found in the Supplementary files.

## Results

### Diet-induced MASH and HCC in mice

**Fig. 1:**
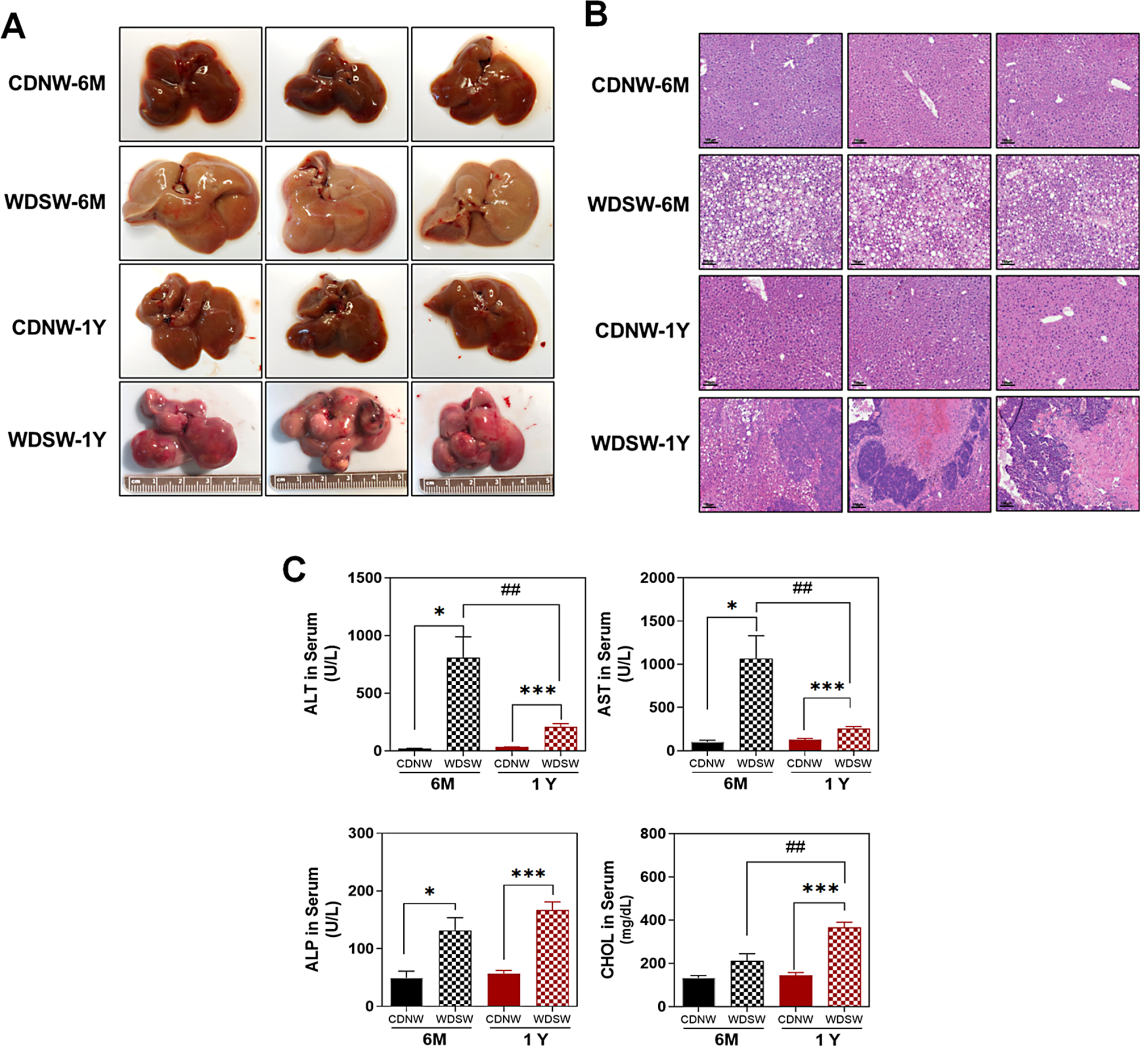
Establishment of MASH-HCC disease progression in DIAMOND mouse model. Wild-type mice with mixed backgrounds of C57BL/6J and 129S1/SvlmJ (male, 21–24 weeks old) were fed with WDSW or CDNW for 6 months (6 M) or 1 year (1Y). **(A)** Representative liver images. **(B)** Representative images of the liver sections with H&E staining. **(C)** Serum levels of liver enzyme levels (ALT, AST, ALP), and total CHOL. Data are presented as the mean ± SEM. Statistical significance relative to the corresponding CDNW group: *p < 0.05, **p < 0.01, ***p < 0.001. Statistical significance relative to WDSW-6 M group: *##p* < 0.01 (*n* = 10–15). WDSW: Western Diet and Sugar Water; CDNW: Control Diet and Normal Water; ALT: Alanine Aminotransferase; AST: Aspartate Aminotransferase; ALP: Alkaline Phosphatase; CHOL: Total Cholesterol

We previously developed a mouse model of MASH-HCC induced by a Western diet with sugar water (WDSW), closely resembling the disease progression observed in human MASH-HCC as described as the DIAMOND mouse model [15]. As shown in Fig. 1A., all mice on WDSW developed fatty liver and MASH after 6 months and HCC after 1 year. Serum levels of ALT and AST were significantly elevated in mice on the WDSW at both 6 months and 1 year, compared to their counterparts on the chow diet with normal water (CDNW). Notably, the AST level in the CDNW-1 year group was significantly higher than that of the CDNW-6 month group. However, the ALP level was only elevated in the HCC group, aligning with the patterns seen in human MASH-HCC. Additionally, the WDSW-1Y (HCC) group, compared to the respective CDNW-1Y group and the MASH (WDSW-6 M) group, as depicted in Fig. 1C, had significantly increased cholesterol (CHOL) levels in the serum. However, serum triglyceride levels did not change (data not shown).

### Dysregulated sphingolipid metabolism in MASH-HCC

**Fig. 2:**
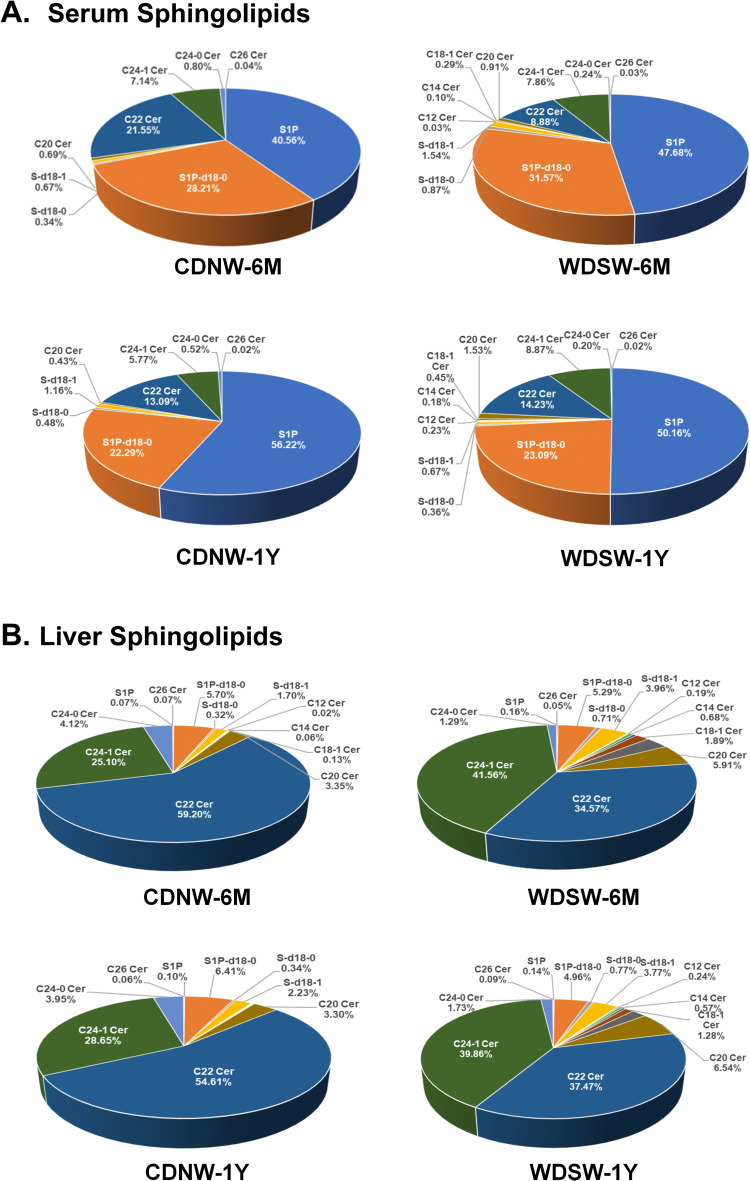
Sphingolipid composition in the serum and liver during MASH-HCC disease progression in DIAMOND mice. Serum and liver tissues were used for a comprehensive sphingolipid analysis with LC-MS/MS. Pie charts showing the sphingolipid profiles from different groups (**A**) in the serum and (**B**) in the liver, expressed as percentages of total sphingolipids

Our previous study reported that changes in bile acid composition and levels were associated with MASH disease progression in animal models and human patients [16, 18]. To further identify possible new biomarkers of MASH-HCC progression, we performed targeted lipidomic analysis for 28 sphingolipids in serum and liver tissues using LC-MS/MS. As shown in Fig. 2A, the predominant sphingolipids in the serum were S1P (d18-1), S1P-d18-0, and ceramide (C22 Cer); whereas in the liver, the major sphingolipids were ceramides (C22 and C24-1) and S1P-d18-0 (Fig. 2B). In the MASH group, the serum composition showed an increase in S1P from 40.56 to 47.68%, a decrease in C22-Cer from 21.55 to 8.88% as well as the presence of C12, C14, and C18-1-Cer which were not detected in controls. In the HCC group, the percentage of S1P decreased from 56.22 to 50.16%, but no significant changes were observed in C22-Cer levels. However, C12, C14, C18-1, C20, and C24-1 ceramides were significantly increased. As shown in Fig. 2B, the percentage of C22-Cer was significantly reduced in both MASH and HCC groups, but the percentage of C24-1 Cer was markedly increased.

**Fig. 3:**
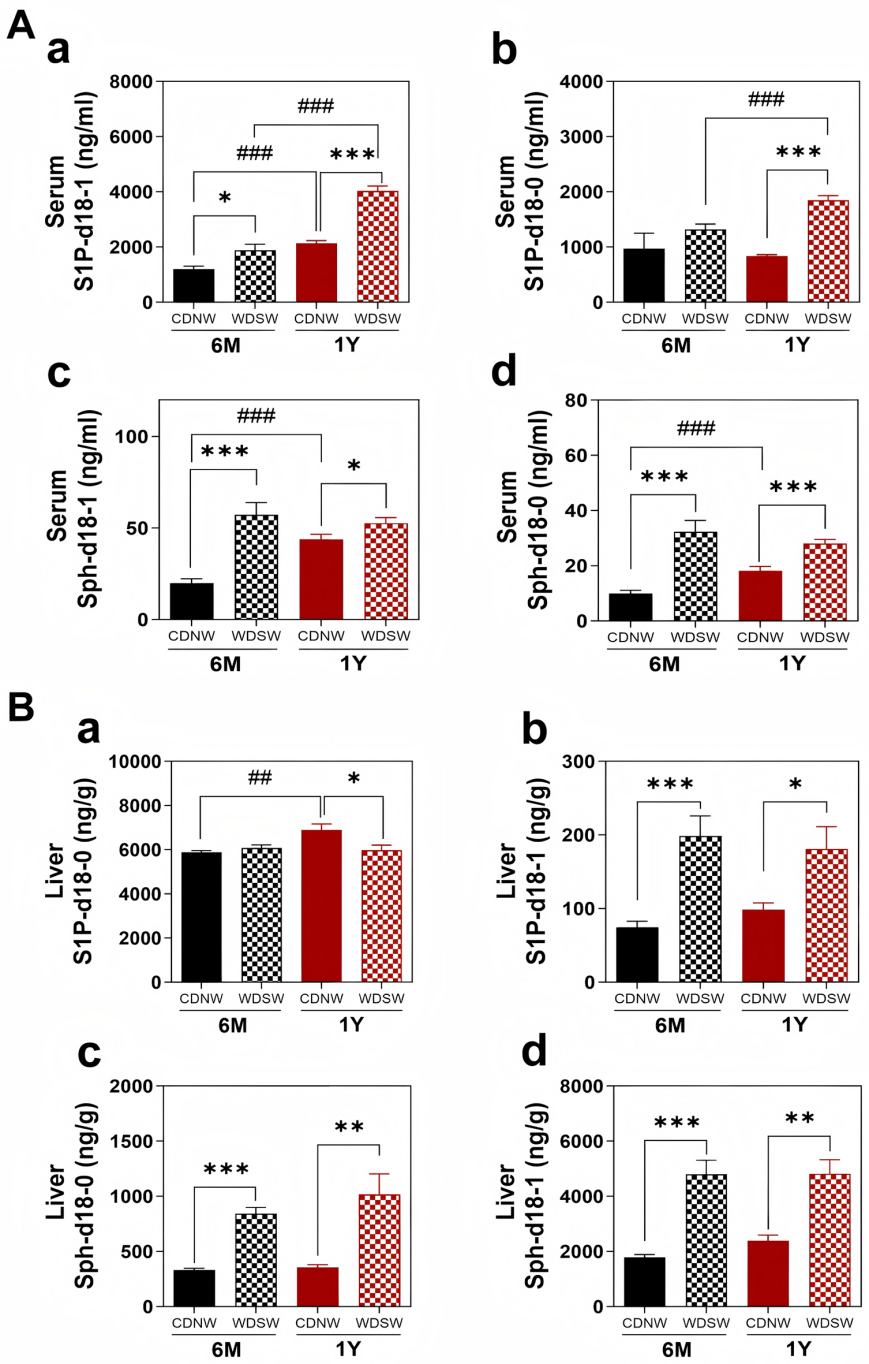
S1P and sphingosine levels in the serum and liver during MASH-HCC disease progression in DIAMOND mice. Serum and liver tissues were processed for sphingolipid analysis using LC-MS/MS. **(A)** S1P-d18-1, S1P-d18-0, Sph-d18-1, and Sph-d18-0 levels in the serum. **(B)** S1P, S1P-d18-0, Sph-d18-1, and Sph-d18-0 levels in the liver. Data are expressed as the mean ± SEM. Statistical significance relative to the corresponding CDNW group: *p < 0.05, **p < 0.01, ***p < 0.001. Statistical significance relative to CDNW-6 M or WDSW-6 M group: *##p* < 0.01, ###*p* < 0.001 (*n* = 10–15)

Further analysis of individual serum sphingolipids in different animal groups during MASH-HCC progression showed that S1P (d18-1) and sphingosine (both sph-d18-1 and sph-d18-0) were significantly increased in both MASH and HCC groups compared to controls. Notably, S1P (d18-1) was the most abundant sphingolipid in the serum, with further elevation observed in the HCC group (Fig. 3Aa). S1P-d18-0 was only increased in the HCC group (Fig. 3Ab). In the liver, S1P-d18-0 was the major sphingolipid. As shown in Fig. 3B, S1P (d18-1) and Sph (sph-d18-1 and sph-d18-0) were increased in both MASH and HCC groups. However, the S1P-d18-0 level was reduced in the HCC group (Fig. 3Bb).

**Fig. 4:**
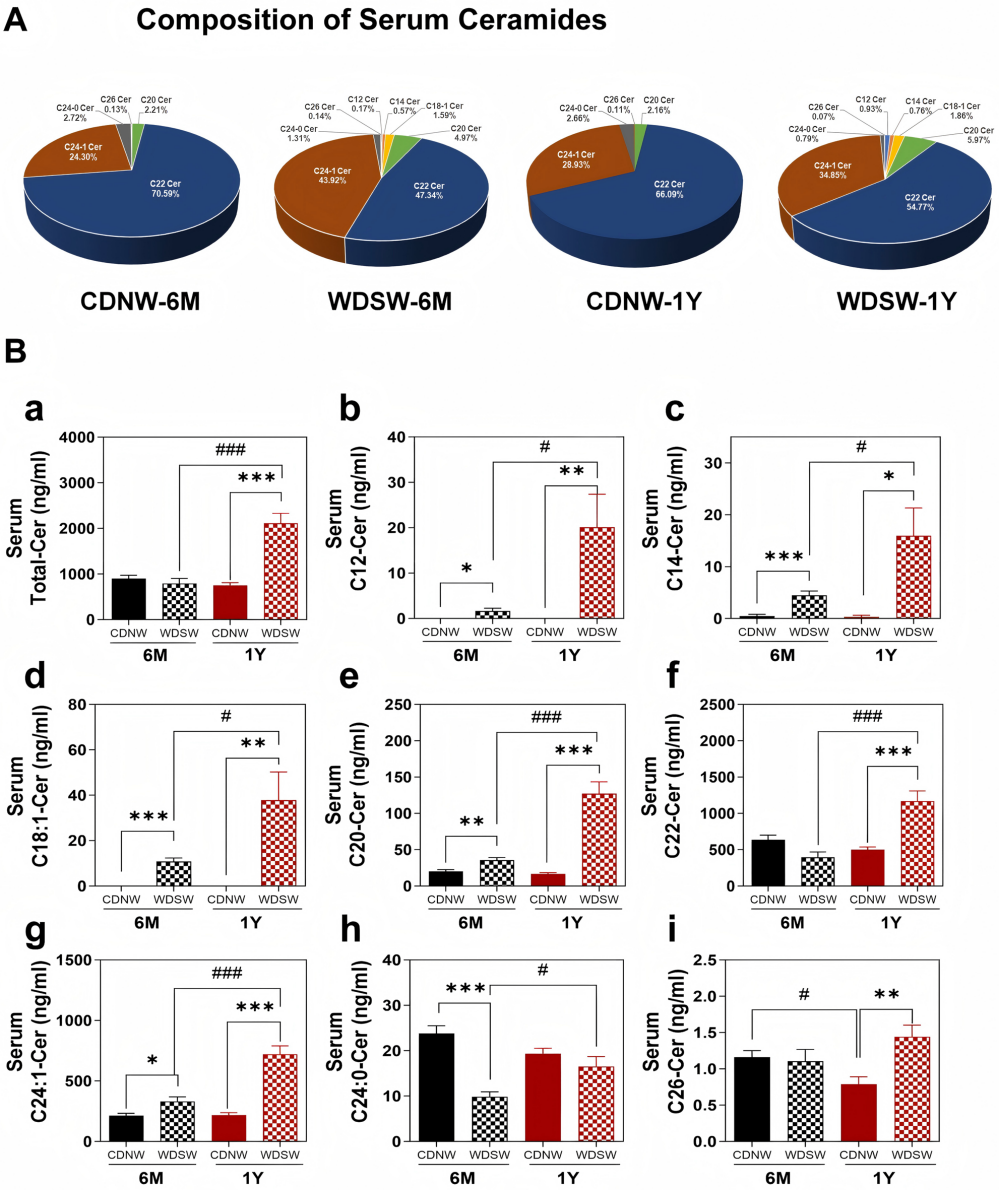
Ceramide composition and levels in the serum during MASH-HCC disease progression in DIAMOND Mice. The serum was processed for ceramide profiles using LC-MS/MS. **(A)** Pie charts showing the ceramide (Cer) composition in the serum from different groups expressed as a percentage of total ceramides. **(B)** Levels of various ceramides in the serum. Data are expressed as the mean ± SEM. Statistical significance relative to the corresponding CDNW group: *p < 0.05, **p < 0.01, ***p < 0.001. Statistical significance relative to CDNW-6 M or WDSW-6 M group: *#p* < 0.05, ###*p* < 0.001 (*n* = 10–15)

**Fig. 5:**
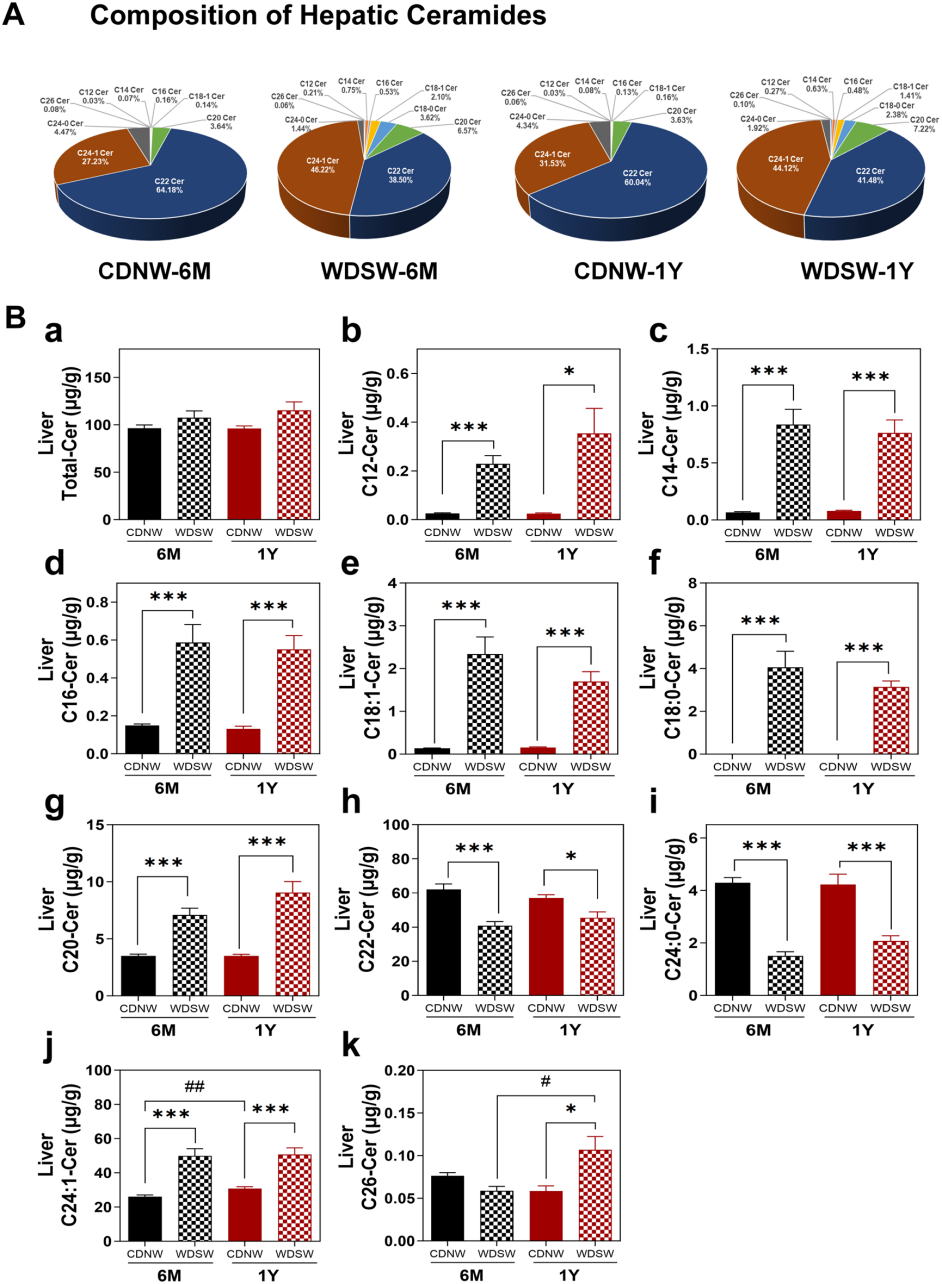
Ceramide composition and levels in the liver during MASH-HCC disease progression in DIAMOND mice. Liver tissues were processed for ceramide profiles using LC-MS/MS. **(A)** Pie charts showing the ceramide (Cer) composition profile in the liver from different groups expressed as a percentage of total Cer. **(B)** Levels of different types of Cers in the liver. Data are expressed as the mean ± SEM. Statistical significance relative to the corresponding CDNW group: *p < 0.05, ***p < 0.001. Statistical significance relative to CDNW-6 M or WDSW-6 M group: *#p* < 0.05, ##*p* < 0.01 (*n* = 10–15)

Analysis of different species of ceramides in both serum and liver indicated significant changes in the composition and levels of individual species. As shown in Fig. 4A, the major species of ceramides in serum were C22 Cer and C24-1 Cer. In the MASH and HCC groups, the percentage of C22-Cer was markedly reduced, but the percentage of C24-1 Cer was increased. Interestingly, the medium-chain ceramides (C12, C14) and C18:1-Cer, which were absent under normal physiological conditions, were detected in both MASH and HCC groups. Specifically, the serum levels of C12, C14, C18:1, C20, and C24:1-ceramides were gradually increased during MASH-HCC progression (Fig. 4Bb, c,d, e,g). However, C22-Cer and C26-Cer, along with total ceramide levels, were only increased in HCC groups (Fig. 4Ba, f, i). In the liver, the percentage of C22-Cer was decreased, but the percentage of C24:1-Cer was increased in both MASH and HCC groups (Fig. 5A). There was no significant change in total ceramide level in the liver (Fig. 5Ba). The hepatic levels of C12, C14, C16, C18:1, C18:0, C20, and C24:1-Cer were upregulated in both MASH and HCC groups (Fig. 5Bb-g&j), whereas the C26-Cer was only increased in HCC groups (Fig. 5Bk). Moreover, C22-Cer and C24:0-Cer levels decreased in the MASH and HCC groups (Fig. 5Bh&i).

These results indicate significant alternations in sphingolipid composition and levels throughout MASH-HCC progression in the mouse model, underlining the potentially critical role of specific sphingolipid species in the MASH-HCC progression.

### Total RNA transcriptome analysis

Total hepatic RNA sequencing identified the DEGs in the MASH-HCC progression in the mouse model. As shown in Fig. S1, the MASH (WDSW-6 M) group had 1042 DEGs (729 upregulated and 313 downregulated) compared to the control group (CDNW-6 M). The HCC (WDSW-1Y) group had 2412 DEGs (1298 upregulated and 1114 downregulated) compared to the CDNW-1Y group. The MASH group had 1852 DEGs (1004 upregulated and 848 downregulated) compared to the HCC group (FC ≥ 1.5; p-value < 0.05). Gene ontology (GO) analysis showed that the top 20 dysregulated gene functions were related to immune cell proliferation, activation, migration, and adhesion, including T cells and myeloid leukocytes in MASH mice compared to control (Fig. S2A). In mice with HCC, the activation of extracellular signal-regulated kinase (ERK), membrane structure and organization, collagen-containing extracellular matrix (ECM), and MHC-protein complex were significantly increased compared to control mice (Fig. S2B). The KEGG pathways analysis showed that the top dysregulated pathways included steroid biosynthesis, phagosome, NF-κB signaling pathway, natural killer cell-mediated cytotoxicity, cytokine and chemokine signaling, and B cell receptor signaling pathway in MASH mice (Fig.S3A). In the HCC group, tight junction, phagosome, ECM-receptor interaction, and cell cycle were among the top dysregulated pathways (Fig.S3B).

To examine the hepatic metabolic changes in MASH-HCC progression, we quantified the key genes involved in the metabolic process using NanoString nCounter with a customized metabolic panel, including sphingolipid metabolism. As shown in Fig.S4A, there were significant changes in many genes related to hepatic lipid metabolism. GO analysis further showed that the major BPs changed in both MASH and HCC groups, including the immune system process, inflammation response, and positive regulation of tumor necrosis factor (TNF) production (Fig.S4B). In the MASH group, the lipid and fatty acid metabolic processes were significantly impacted. GO-cellular component (GO-CC) analysis showed during MASH-HCC progression, plasma membrane, cell surface, cytoplasm, and extracellular space were markedly changed (Fig.S4C). GO-molecular function (GO-MF) analysis showed that protein binding, identical protein binding, and macromolecular binding were top tree-impacted MF in MASH and HCC groups (Fig.S4D). KEGG pathway analysis showed that the sphingolipid signaling pathway was significantly changed, especially in the HCC group (Fig.S4E).

**Fig. 6:**
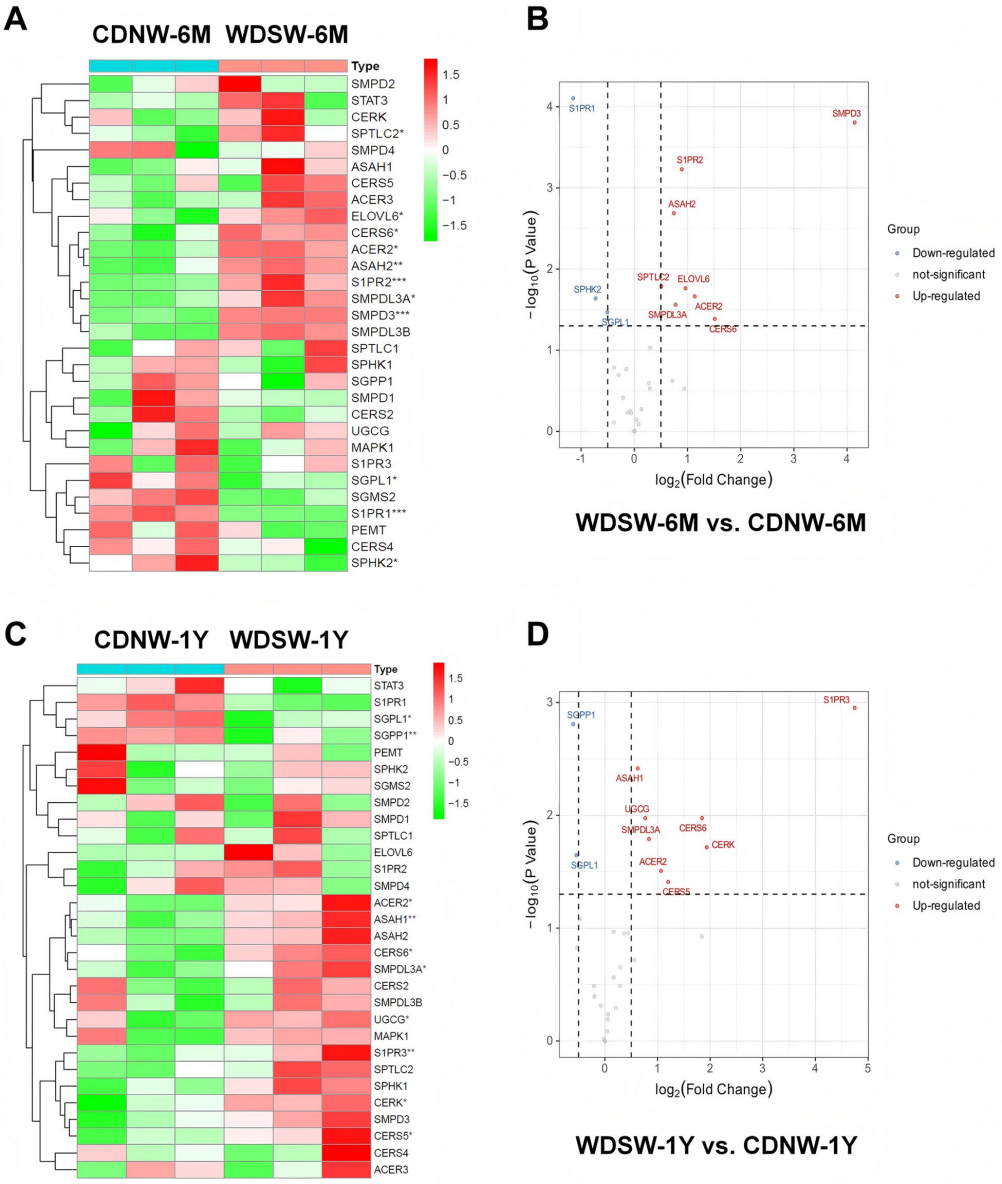
Hepatic metabolic gene profile during MASH-HCC disease progression in DIAMOND mice. NanoString nCounter mouse metabolic mRNA panel with a customized sphingolipid metabolic panel was used to profile the significant changes in key genes related to both hepatic lipid and sphingolipid metabolism in MASH-HCC progression. Liver RNA samples from each group (*n* = 3) were used. DEGs were identified in different groups using FC and p-values (FC ≥ 1.5 and p-value < 0.05). **(A)** Hierarchical clustering heatmaps display changes in sphingolipid metabolism-related genes between WDSW-6 M and CDNW-6M. **(B)** Volcano plots highlight major upregulated and downregulated genes in WDSW-6 M compared to the CDNW-6 M group. **(C)** Hierarchical clustering heatmaps display changes in sphingolipid metabolism-related genes between WDSW-1Y and CDNW-1Y. **(D)** Volcano plots highlight major upregulated and downregulated genes in WDSW-1Y compared to the CDNW-1Y group. Statistical significance relative to CDNW group: *p < 0.05, **p < 0.01, ***p < 0.001 (*n* = 3, male). DEG: Differentially Expressed Gene; FC: Fold Change

Further analysis of the major genes involved in sphingolipid metabolism and signaling showed that sphingosine-1-phosphate receptor 1 (S1pr1), sphingosine kinase 2 (Sphk2) and sphingosine-1-phosphate lyase 1 (Sgpl1) were significantly downregulated, but sphingomyelin phosphodiesterase 3 (Smpd3), Smpdl3a, S1pr2, neutral ceramidase (Asah2), ceramide synthase 6 (Cers6), alkaline ceramidase 2 (Acer2), and elongation of very long chain fatty acids protein 6 (Elovl6) were significantly upregulated in MASH mice (Fig. 6A&B). In the MASH-HCC mouse model, sphingosine-1-phosphate phosphatase 1 (Sgpp1) and Sgpl1 were significantly downregulated, while Cers6, Acer2, Cers5, S1pr3, Cerk, Asah1, Smpdl3a, and glucosylceramide synthase (Ugcg) were significantly upregulated (Fig. 6C&D). As shown in Fig.S5, Cers6, Smpdl3a, and Acer2 were upregulated, and Sgpl1 was downregulated in both MASH and HCC groups.

### Single nuclei RNA-seq reveals cell-specific changes in the sphingolipid metabolism in MASH-HCC

To delineate cell type-specific alterations within sphingolipid metabolism pathways, we examined the recently published snRNA-seq data (GSE225381) from our DIAMOND mouse model [19]. Within this data set, we identified all hepatic cell populations using cell-type-specific marker genes, including hepatocytes (57%), macrophages (12.87%, monocyte-derived macrophage and Kupffer cells (KCs), MdMQs and KClike), endothelial cells (ECs) (12.39%), hepatic stellate cells (HSCs) (8.45%, HSCs), cholangiocytes (1.68%), lymphocytes (4.27%, Lyms), B cells (2.36%), stromal cells (0.91%), and dendritic cells (0.56%, DCs). Notably, the percentage of hepatocytes and ECs was increased in the HCC group. Conversely, the KC-like cell population exhibited an increase in the pre-tumor group compared to the control group, followed by a decrease in the HCC group relative to the pre-tumor group. Lyms percentage of cells and cell counts were significantly reduced in the HCC group vs. pre-tumor group, not the pre-tumor group vs. control (Fig. S6). Further analysis of each group unveiled a distinct sub-population of hepatocytes in the pre-tumor and HCC groups compared to the control group (Fig.S7). During MASH-HCC disease progression, there was a notable depletion of peri-portal hepatocytes alongside an augmentation of mid-zone and peri-central hepatocytes. Interestingly, there was a marked increase in proliferative hepatocyte clusters in the HCC group (Fig. S7B). As shown in Fig.S8, the percentages of MdMQs were increased in the pre-tumor and HCC groups. However, the healthy KC1 cluster was lost entirely, while tumor-associated macrophages (TAM) were increased in HCC. Additionally, a subpopulation of MASH-associated macrophages (MAM) was identified in the pre-tumor group (Fig.S8D). The number of lyms identified in this snRNAseq data set was limited. As illustrated in Fig.S9, the numbers of CD8s and Tregs were increased in the pre-tumor group but not the HCC group. Conversely, the numbers of CD4 + T cells and natural killer cells (NKs) were reduced in both the pre-tumor and HCC groups.

**Fig. 7:**
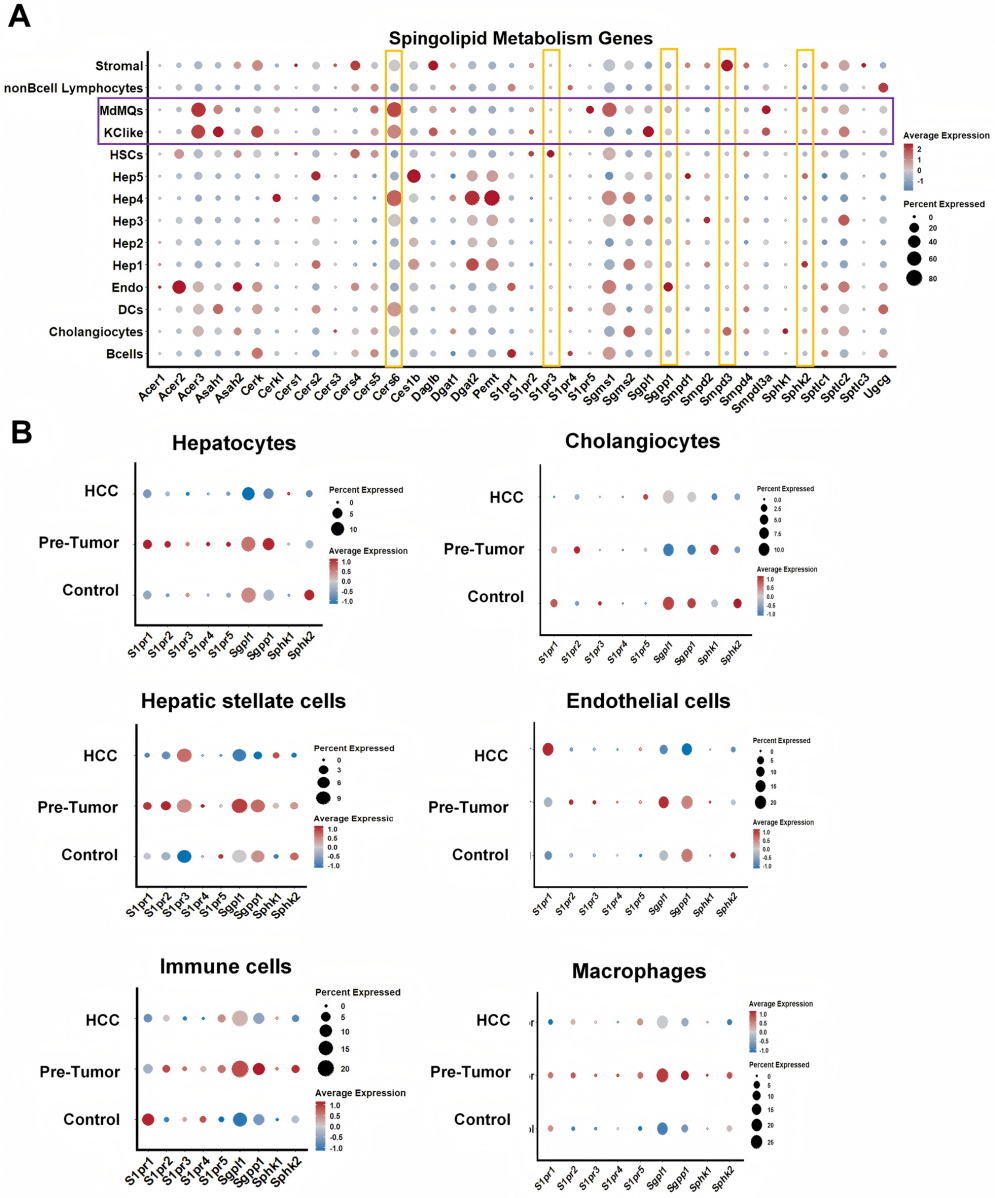
Expression of sphingolipid metabolism-related genes in different hepatic cells. The GEO data set (GSE225381) of snRNAseq of DIAMOND mice was downloaded and analyzed. **(A)** Dot plot showing the percentage of cells expressing specific genes involved in sphingolipid metabolism and their relative expression levels in each cell type. **(B)** Dot plots for different cell types, showing the percentage of cells expressing S1pr1-5, Sgpl1, Sgpp1, *Sphk1*, and *Sphk2*, and their relative expression levels under different conditions

To delve into the cell-type-specific changes in hepatic sphingolipid metabolism pathways, we initially examined the expression profiles of key genes involved in ceramide and S1P synthesis, degradation, and signaling across distinct hepatic cell types. As shown in Fig. 7A, these genes exhibited differential expression patterns across various hepatic cells. Cers6 was highly expressed in a subset of hepatocytes (Hep4) and macrophages, particularly in MdMQs. S1pr1 was predominantly detected in B cells and ECs, while S1pr2 was mainly detected in cholangiocytes, HSCs, MdMQs, and KC-like cells. Notably, S1pr3 exhibited pronounced expression in HSCs, and Smpd3 showed elevated expression levels in stromal cells and cholangiocytes. Sphk2 had higher expression in hepatocytes, cholangiocytes, and macrophages. Furthermore, Sphk2 expression was significantly downregulated across all hepatic cells in the HCC group (Fig. 7B). In hepatocytes, S1PRs, especially S1pr1 and S1pr2, were only up-regulated in the pre-tumor group. Conversely, in cholangiocytes, S1pr1 was downregulated in pre-tumor and HCC groups. In HSCs, S1pr1-3 was upregulated in the pre-tumor group, but only S1p3 was further upregulated in the HCC group. Interestingly, in ECs, S1pr1 was upregulated in the HCC group. However, S1pr1 was downregulated in the HCC group in immune cells and macrophages. Of particular interest, Sgpl1 and Sgpp1, key enzymes involved in the metabolism of S1P, were significantly downregulated across all hepatic cells in the HCC group but increased in hepatocytes, HSCs, ECs, immune cells, and macrophages in the pre-tumor group.

Analysis of the key enzymes involved in ceramide synthesis and metabolism revealed a significant upregulation of Cers6 in hepatocytes, cholangiocytes, HSCs, and immune cells in both pre-tumor and HCC groups. Additionally, Cers6 was upregulated in ECs and macrophages in the pre-tumor group (Fig.S10). Moreover, Acer2 expression was elevated in HSCs and ECs. Acer3 expression was significantly upregulated in cholangiocytes, immune cells, and macrophages in the HCC group. Furthermore, sphingomyelin synthase 1 (Sgms1) and Sgms2 were differentially expressed in different hepatic cell types during HCC progression. Specifically, Sgms1 was upregulated in hepatocytes, ECs, and macrophages of the HCC group but downregulated in cholangiocytes and HSCs. Conversely, Sgms2 expression was downregulated in hepatocytes, HSCs, ECs, immune cells, and macrophages of both pre-tumor and HCC groups but upregulated in cholangiocytes of the HCC group. Interestingly, Sptlc2 (serine palmitoyltransferase, long chain base subunit 2), a critical subunit of the enzyme serine palmitoyltransferase, was upregulated in hepatocytes, immune cells, and macrophages but downregulated in cholangiocytes, HSCs, and ECs in HCC group. The downregulation of S1pr1 and upregulation of Cer6 and Sptlc2 were further validated by qPCR (Fig.S10B). The graphical summary visually outlines the key sphingolipid genes across different cell types during MASH-HCC progression (Fig.S11).

### Profiling and establishing a sphingolipid gene signature in human HCC patients

**Fig. 8:**
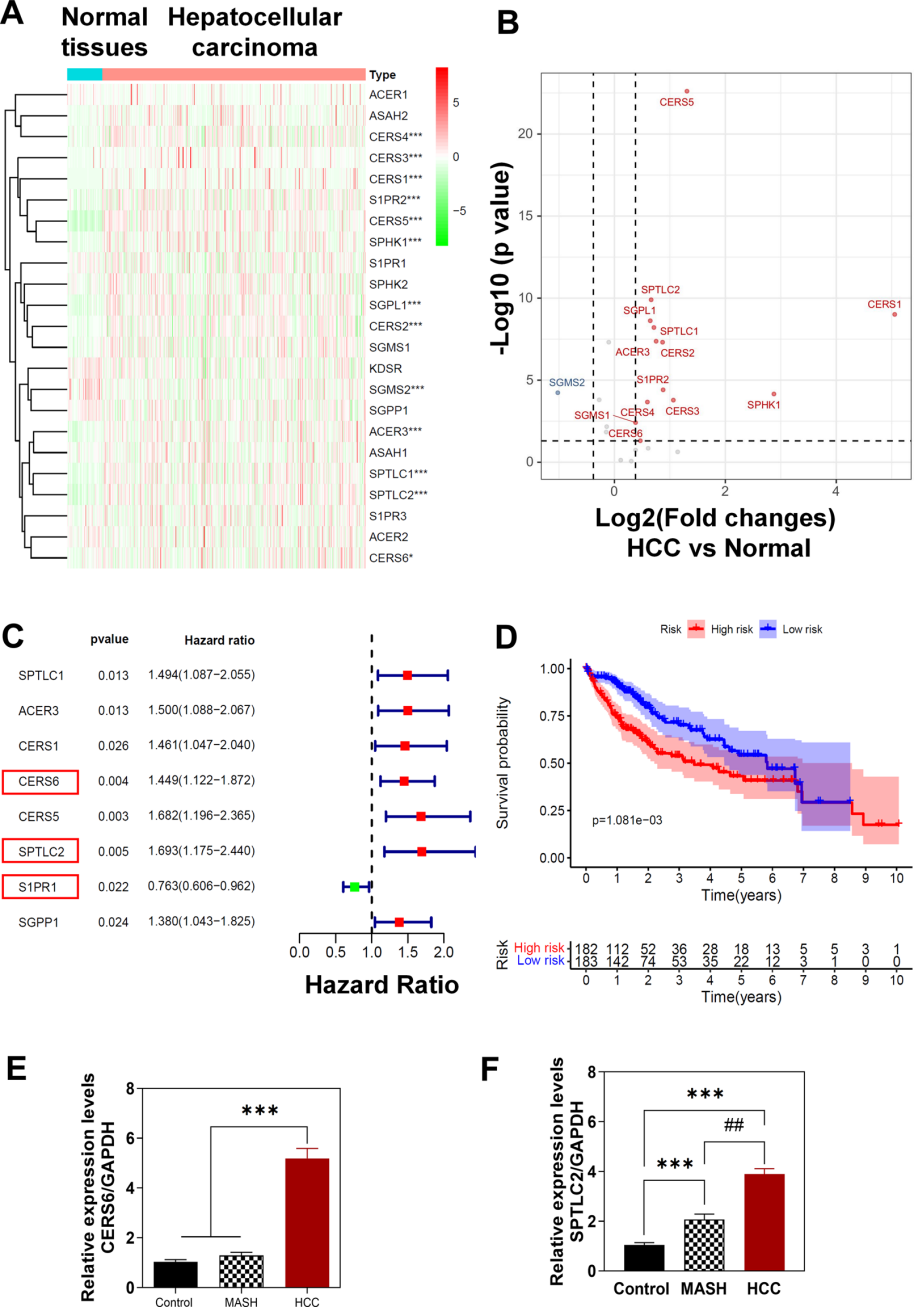
Analysis of sphingolipid metabolism-related genes and prognostic indicators in the TCGA-LIHC Cohort. **(A)** Hierarchical clustering heatmaps display the expression of sphingolipid metabolism-related genes in the TCGA-LIHC cohort. **(B)** Volcano plots highlight differences in sphingolipid metabolism-related gene expression in the TCGA-LIHC cohort. **(C)** A forest plot shows univariate and multivariate Cox regression analysis, identifying prognostic sphingolipid metabolism-related genes correlated with overall survival in the TCGA-LIHC cohort. The hazard ratio of each identified gene was calculated for risk score assessment. **(D)** The Kaplan-Meier (KM) survival curves for high- and low-risk groups demonstrate significant differences with P value < 0.05. **(E-F)** Relative mRNA levels of two key genes involved in sphingolipid metabolism in the liver samples from MASH and HCC patients, including CERS6 and SPTLC2 were determined by qRT–PCR and normalized with Gapdh as an internal control. Data are expressed as the mean ± SEM. Statistical significance relative to control: ***p < 0.001. Statistical significance relative to MASH: ##*p* < 0.01; ###*p* < 0.001 (*n* = 8)

To assess the translational relevance of our findings from DIAMOND mouse models to human MASH-HCC, we analyzed the TCGA-LIHC dataset, focusing on genes related to sphingolipid metabolism. As shown in Fig. 8A-B, the expression levels of ACER3, CERS1, CERS2, CERS3, CERS4, CERS5, CERS6, S1PR2, SGMS1, SGPL1, SPHK1, SPTLC1, and SPTLC2 were significantly upregulated, while SGMS2 was significantly downregulated compared to control. Through univariate Cox analysis, we identified several sphingolipid metabolism genes linked to overall survival (OS) in the TCGA-LIHC cohort, including SPTLC1, ACER3, CERS1, CERS6, CERS5, SPTLC2, S1PR1, and SGPP1 (Fig. 8C). A subsequent multivariate Cox analysis was conducted to further narrow down these genes into a prognostic signature comprising three genes: CERS6, SPTLC2, and S1PR1. CERS6 and SPTLC2 were classified into a high-risk group with higher hazard ratios (HR > 1) and higher expression in tumor tissues, but S1PR1 exhibited a protective effect (HR < 1) and lower expression in malignant tissues. A prognostic model based on multivariate Cox analysis was developed, and the risk score was calculated using specific coefficients. The risk score was calculated by the formula: risk score= (0.2934 × CERS6) + (0.4468 × SPTLC2) + (-0.4037 × S1PR1). The TCGA-LIHC cohort was divided into high-risk (*n* = 182) and low-risk (*n* = 183) groups based on the median risk score. Kaplan-Meier analysis demonstrated that the high-risk group had significantly poorer OS than the low-risk group (*p* = 1.081e-03) (Fig. 8D). Furthermore, we validated the expression of CERS6 and SPTLC2 in human MASH and HCC patient samples using qPCR. As shown in Fig. 8E-F, both CERS6 and SPTLC2 were markedly upregulated in HCC patients, highlighting their potential as diagnostic and prognostic markers in human MASH-HCC.

## Discussion

The global rise in obesity, type 2 diabetes, and metabolic syndrome has led to a significant increase in MASH-HCC [20–23]. The DIAMOND mouse model closely mimics human disease progression. Mice developed steatosis in 8 weeks, early MASH in 16 weeks, severe MASH and fibrosis in 26 weeks, and HCC in 40 to 52 weeks [15]. Recent studies have reported that circulating and hepatic sphingolipids were changed in both HFD-fed mouse models [24]. However, the HFD-fed mouse model in this study only represents hepatic steatosis and early MASH [24]. The changes in sphingolipids in MASH-HCC disease progression have not been well characterized. The present study provides comprehensive insights into the dysregulation of sphingolipid metabolism pathways in murine models and human MASH-HCC patients. Our findings underscore the pivotal roles of sphingolipids in MASH-HCC pathogenesis and progression, highlighting potential diagnostic and prognostic biomarkers for MASH-HCC.

The MASH-HCC mouse model exhibits significant alterations in the expression profiles of key enzymes involved in ceramide synthesis and metabolism across various hepatic cell types. Our targeted lipidomic analysis identified marked changes in sphingolipid composition and levels during the disease progression of MASH to HCC. The key findings included a significant increase in S1P and specific ceramides in the serum and the liver (Figs. 2, 3, 4 and 5). The serum level of total ceramides was increased in HCC, along with a distinct composition and marked increase of different ceramide species compared to MASH. These findings suggest that these ceramides are crucial in promoting MASH-HCC progression.

Total RNAseq, snRNAseq, and NanoString gene profiles identified important signaling pathways, cellular components, and a complex interplay between metabolic and immune dysfunction in MASH-HCC progression. Analysis of snRNAseq provided a detailed view of cell type-specific changes in gene expression related to sphingolipid metabolisms, such as Cers6 and S1pr1. These results highlighted the importance of cell-specific metabolic regulation of sphingolipids in MASH-HCC. It has been reported that increased ceramides promote insulin resistance in obesity [25]. Six ceramide synthases have been identified, each responsible for generating different acyl-chain ceramides [26]. It has been reported that the biological and pathological activities of ceramides are impacted by the length and nature of the acyl chain. C16:0-Cer and C18:0-Cer are important nutritional signaling molecules, but C24:0 or C24:1 are not [27]. CerS6 is mainly responsible for C16:0-Cer and C14-Cer synthesis, and its expression is upregulated in obesity. Liver-specific deletion of CerS6 prevents insulin resistance and hepatic steatosis [25, 28]. Recent studies also found that CerS6 upregulation is associated with alcohol-associated liver disease and cardiovascular disease [29, 30]. In hepatocytes, overexpression of CerS6 reduced the protein Insig-1 (Insulin-induced gene 1), resulting in increased SREBP-1c (sterol regulatory element binding protein 1c) cleavage and subsequent enhanced lipogenesis [31]. CerS6-derived C16:0 Cer is lipotoxic, binds to mitochondrial fission factor, and induces mitochondrial damage [32, 33]. It has also been reported that CerS6-derived C14:0 Cer is linked to insulin resistance and obesity [34]. Our snRNAseq data analysis showed that CerS2 is the predominant CerS in the hepatocytes under normal physiological conditions. The expression level of CerS6 was significantly upregulated in hepatocytes, cholangiocytes, and immune cells in both pre-tumor and HCC groups (Fig. S10). Chronic inflammation, marked by elevated cytokines like TNF-α, IL-6, and IL-1β, activates pathways such as NF-κB, which regulate sphingolipid biosynthesis [35]. This results in the upregulation of sphingomyelinases, leading to increased ceramide levels that promote apoptosis, inflammation, and fibrosis, thereby advancing HCC progression. Previous studies reported that activation of CerS6 was associated with TNF-α secretion and hepatic inflammation in HFD-fed mouse models [36]. Consistent with our lipidomic analysis, the major products of CerS6, C14-Cer, and C16-Cer were upregulated in both MASH and HCC mice (Fig. 5). In serum, the total ceramide level was increased only in the HCC group, which was accompanied by marked increase of C12-Cer, C14-Cer, C18-20 Cer.

Sptlc2 is one of the major SPT subunits and was upregulated in hepatocytes, cholangiocytes, and immune cells in both pre-tumor and HCC groups (Fig.S10). ER stress signaling is a well-established player in MASH-HCC [37]. It has been reported that ER stress-induced upregulation of Sptlc2 *via* X-box binding protein 1-mediated transcriptional activation increased the *de novo* biosynthesis of ceramide [38]. This exacerbates hepatic inflammation and insulin resistance, further contributing to disease progression.

Our previous studies reported that bile acid-induced activation of S1PR2 modulated hepatic SphK2 activity, which contributed to regulating hepatic lipid metabolism. Dysregulation of hepatic lipid metabolism, a hallmark of MASH, increases ceramide accumulation, promoting lipotoxicity, insulin resistance, steatosis, and eventually HCC. Deletion of either S1PR2 or SphK2 significantly increased hepatic steatosis and inflammation [39, 40]. We also reported that conjugated bile acid-induced activation of S1PR2 promoted the growth of cholangiocarcinoma cells and esophageal adenocarcinoma cells [41, 42]. Our total RNA transcriptome and NanoString gene profiling showed upregulation of S1PR2 and downregulation of SphK2 in MASH mice (Fig. 6 &S4-5). Consistently, snRNAseq data also identified upregulation of S1PR2 and downregulation of SphK2 across all different hepatic cells in the pre-tumor group and HCC, respectively (Fig. 7B&S11). Upregulation of S1PR1 in ECs has been reported to promote angiogenesis and progression of HCC [43]. Although total S1PR1 levels were reduced in MASH and HCC mouse models (Fig.S10), its expression level was significantly upregulated in hepatic ECs (Fig. 7B) along with an increase of its high-affinity natural agonist S1P-d18-1 in the serum and the liver (Fig. 3). S1PR1 also plays a vital role in directing T-cell migration and supporting T-cell survival [44]. Inhibition of S1PR1 enhanced cancer cell migration in bladder carcinoma [45]. Human TCGA HCC data analysis also showed an association of high risk with lower S1PR1 expression (Fig. 8).

Our results suggest that the serum sphingolipid profiles could be a diagnostic and prognostic biomarker for MASH-HCC progression. Hepatic cell-type-specific targeting of sphingolipid metabolic pathways represents a promising therapeutic approach for preventing and treating HCC.

## Electronic supplementary material

Below is the link to the electronic supplementary material.

Supplementary Material 1

Supplementary Material 2

Supplementary Material 3

Supplementary Material 4

Supplementary Material 5

Supplementary Material 6

Supplementary Material 7

Supplementary Material 8

Supplementary Material 9

Supplementary Material 10

Supplementary Material 11

Supplementary Material 12

## Data Availability

Detailed methods and datasets generated and/or analyzed during the current study are available in the Supplementary file.

## Abbreviations

Acer2: Alkaline ceramidase 2
ALP: Alkaline phosphatase
ALT: Alanine aminotransferase
Asah2: Neutral ceramidase
AST: Aspartate aminotransferase
BP: Biological process
CC: Cellular component
CDNW: Control diet and normal water
CerS: Ceramide synthase
Cers6: Ceramide synthase 6
CHO: Cholangiocyte
CHOL: Total cholesterol
CI: Confidence interval
DAVID: Database for Annotation, Visualization, and Integrated Discovery
DEG: Differentially expressed gene
DIAMOND: A diet-induced MASLD animal model
EC: Endothelial cell
ECM: Extracellular matrix
Elovl6: Elongation of very long chain fatty acids protein 6
ERK: Extracellular signal-regulated kinase
FC: Fold change
GO: Gene Ontology
GSEA: Gene Set Enrichment Analysis
H&E: Hematoxylin and eosin
HCC: Hepatocellular carcinoma
Hep: Hepatocyte
HR: Hazard ratio
HSC: Hepatic stellate cell
KC: Kupffer cell
KEGG: Kyoto Encyclopedia of Genes and Genomes
k-NN: k-nearest neighbors
LC-MS/MS: Liquid chromatography/tandem mass spectrometric
Lym: Lymphocyte
MAM: MASH-associated macrophages
MASH: Metabolic dysfunction-associated steatohepatitis
MASLD: Metabolic dysfunction-associated steatotic liver disease
MdMQ: Monocyte-derived macrophage
MF: Molecular function
MyoF: Myofibroblast
NKs: Natural killer cells
NT: Non-tumoral
PCA: Principal component analysis
S1pr1: Sphingosine-1-phosphate receptor 1
scRNA: Seq-single-cell RNA sequencing
Sgms1: Sphingomyelin synthase 1
Sgpl1: Sphingosine-1-phosphate lyase 1
Sgpp1: Sphingosine-1-phosphate phosphatase 1
Smpd3: Sphingomyelin phosphodiesterase 3
SNN: Shared nearest neighbor
snRNA: Seq-single-nucleus RNA sequencing
Sphk2: Sphingosine kinase 2
Sptlc2: Serine palmitoyltransferase long chain base subunit 2
TAM: Tumor-associated macrophages
TCGA: The Cancer Genome Atlas
Ugcg: Glucosylceramide synthase
UMAP: Uniform Manifold Approximation and Projection
WDSW: Western Diet and Sugar water
