# Supplementary figures and images for: Transcriptomics, lipidomics, and single-nucleus RNA sequencing integration: exploring sphingolipids in MASH-HCC progression
Source: Cell Biosci. 2025 Mar 8;15:34. doi: 10.1186/s13578-025-01362-5 (PMC11890728; doi:10.1186/s13578-025-01362-5)

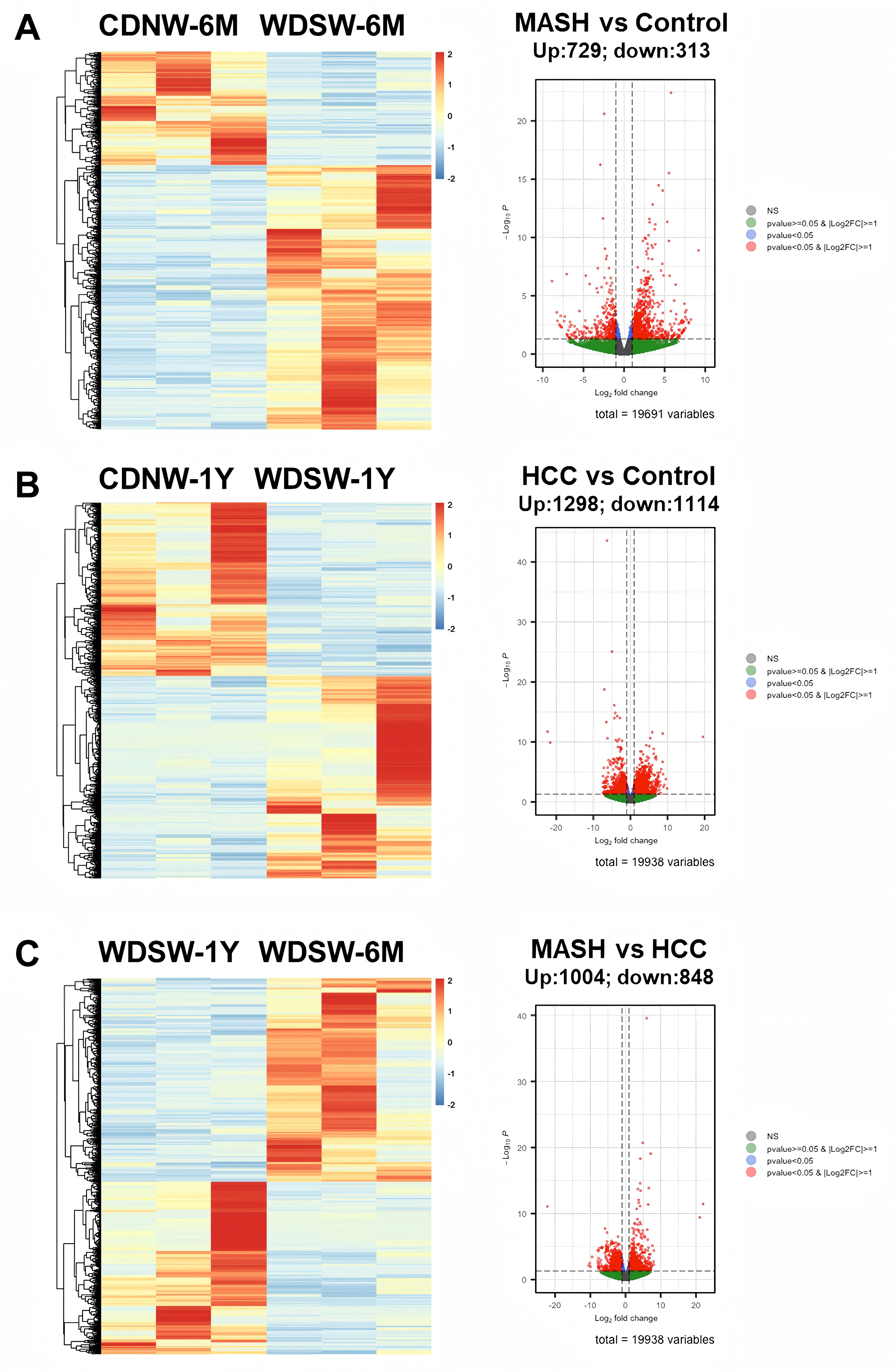

Supplement: Supplementary file 1 — Supplementary Material 1 [file 13578_2025_1362_MOESM1_ESM.tiff]

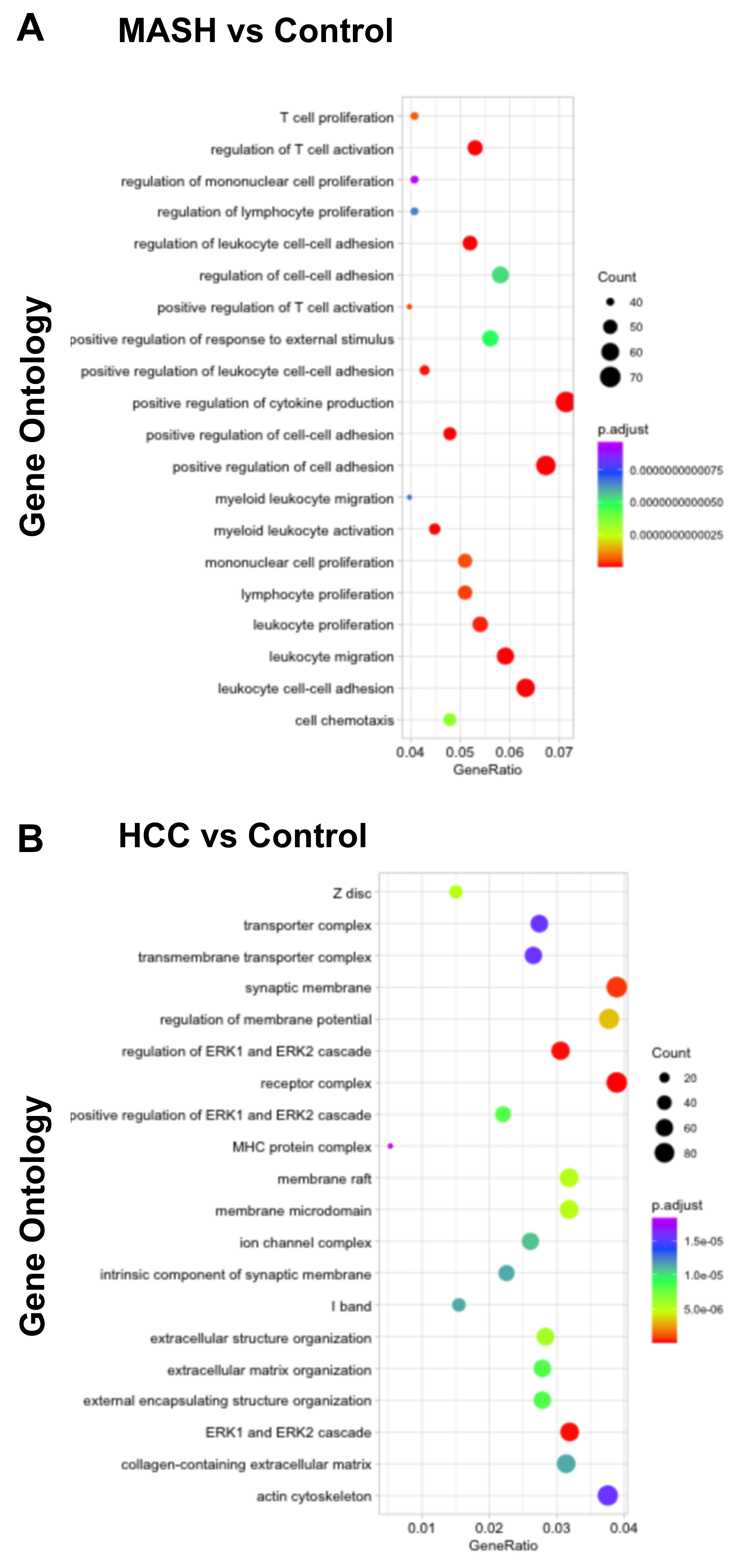

Supplement: Supplementary file 2 — Supplementary Material 2 [file 13578_2025_1362_MOESM2_ESM.tiff]

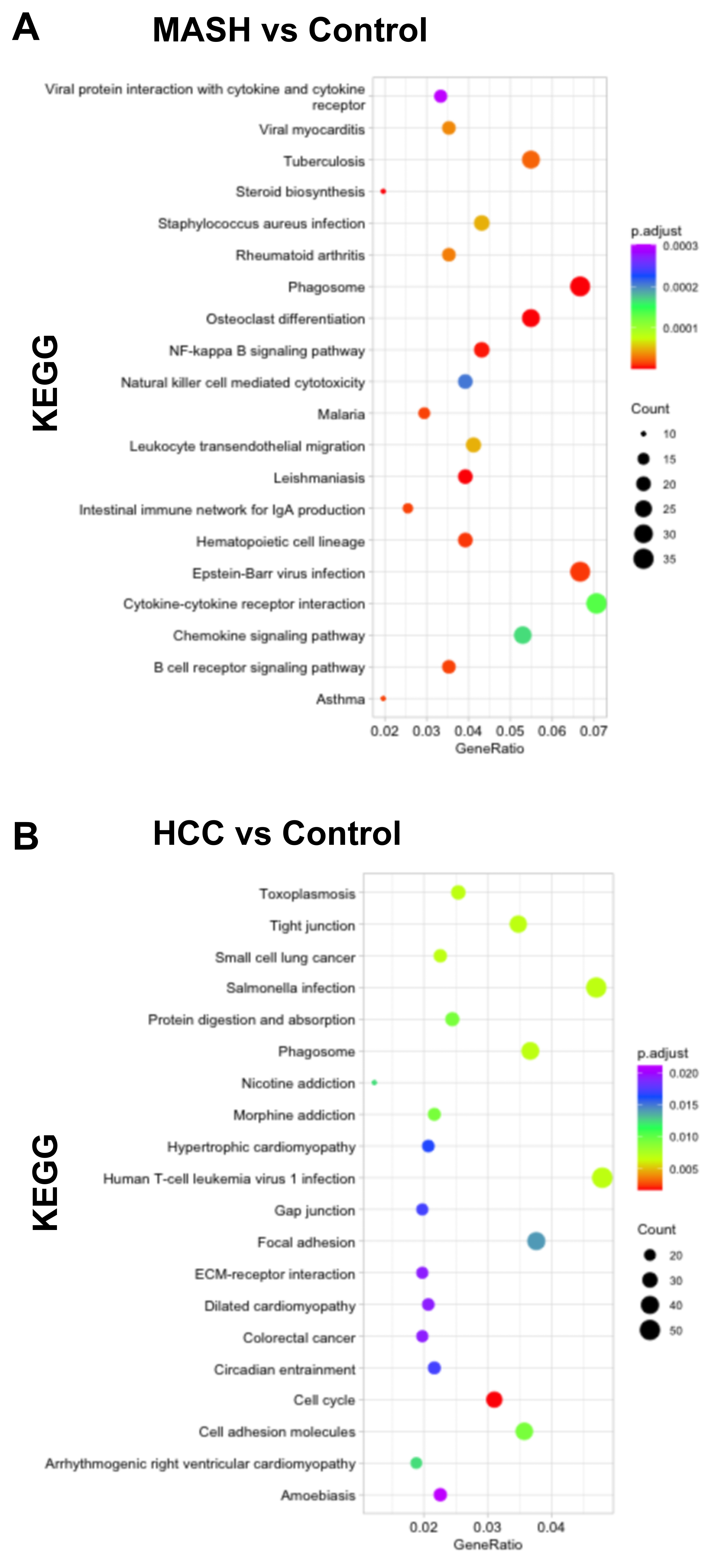

Supplement: Supplementary file 3 — Supplementary Material 3 [file 13578_2025_1362_MOESM3_ESM.tiff]

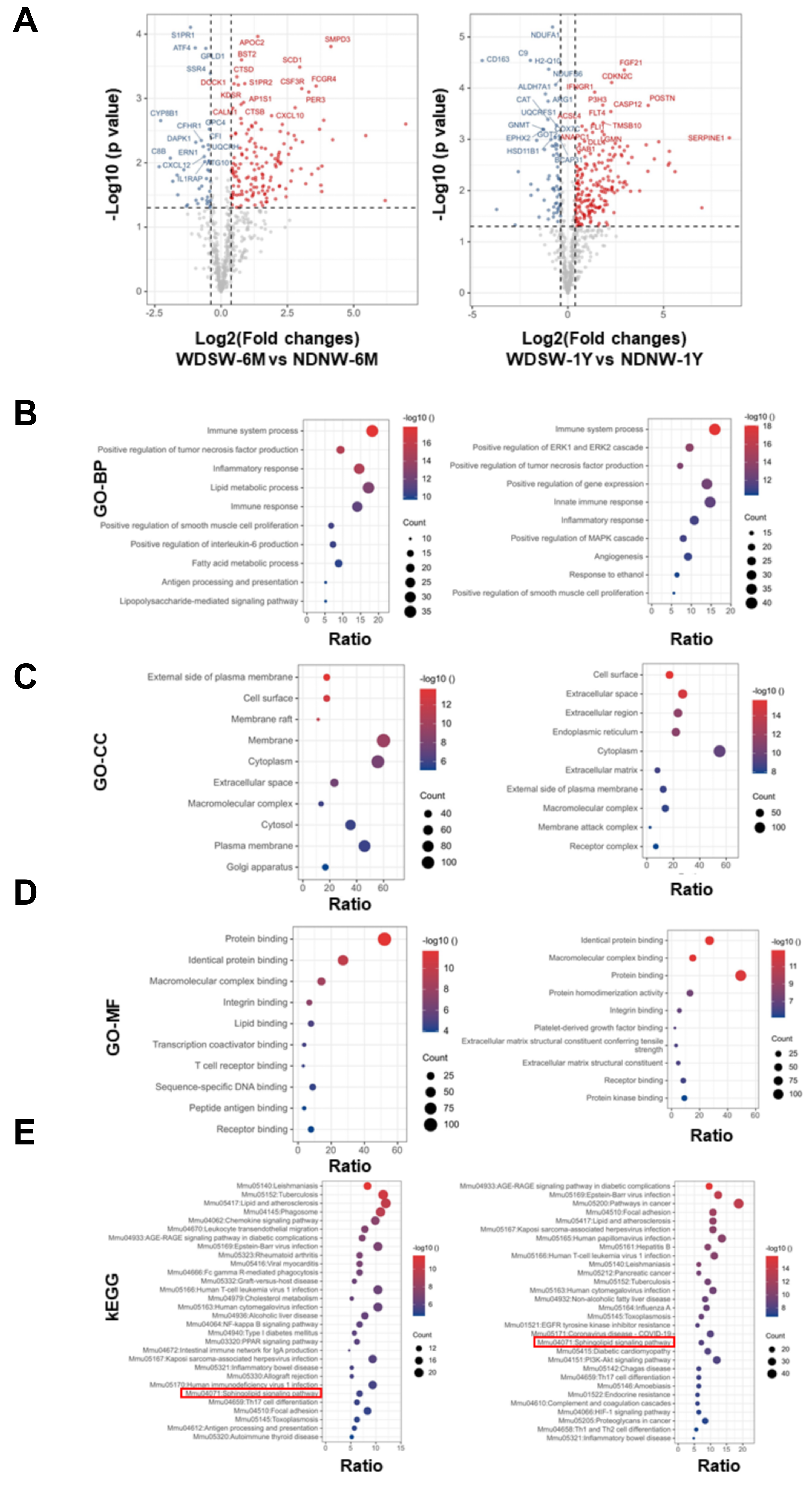

Supplement: Supplementary file 4 — Supplementary Material 4 [file 13578_2025_1362_MOESM4_ESM.tiff]

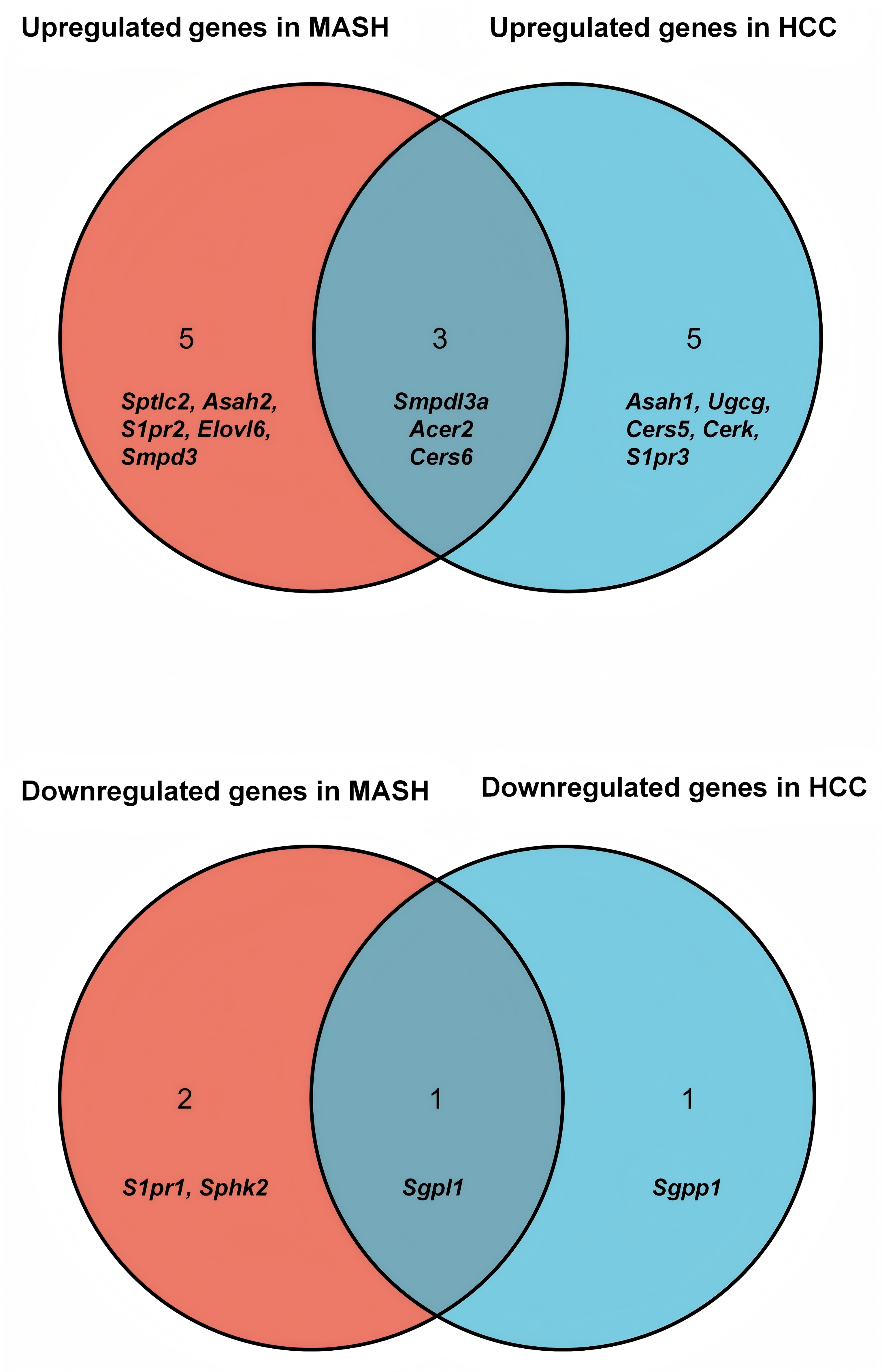

Supplement: Supplementary file 5 — Supplementary Material 5 [file 13578_2025_1362_MOESM5_ESM.tiff]

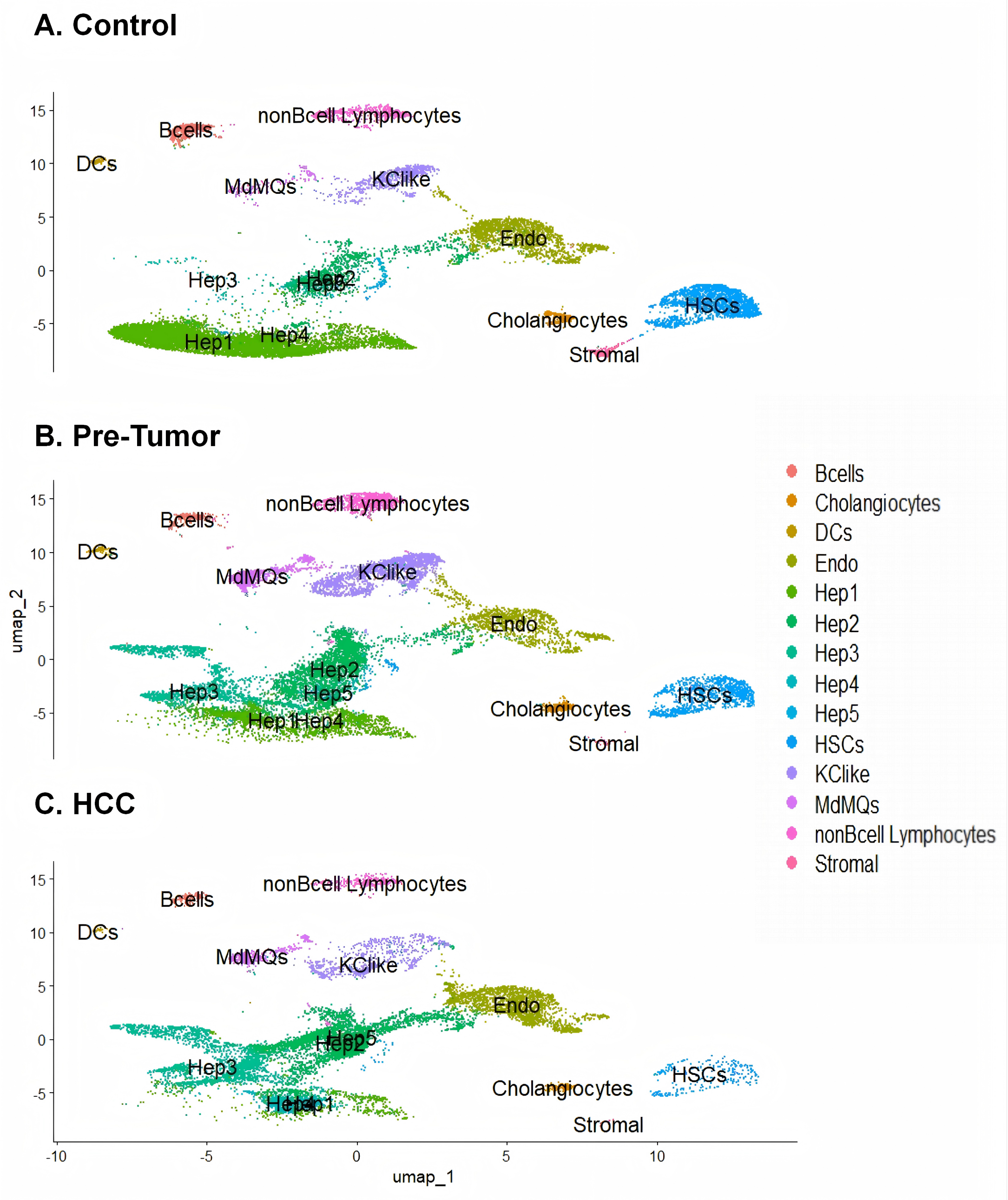

Supplement: Supplementary file 6 — Supplementary Material 6 [file 13578_2025_1362_MOESM6_ESM.tiff]

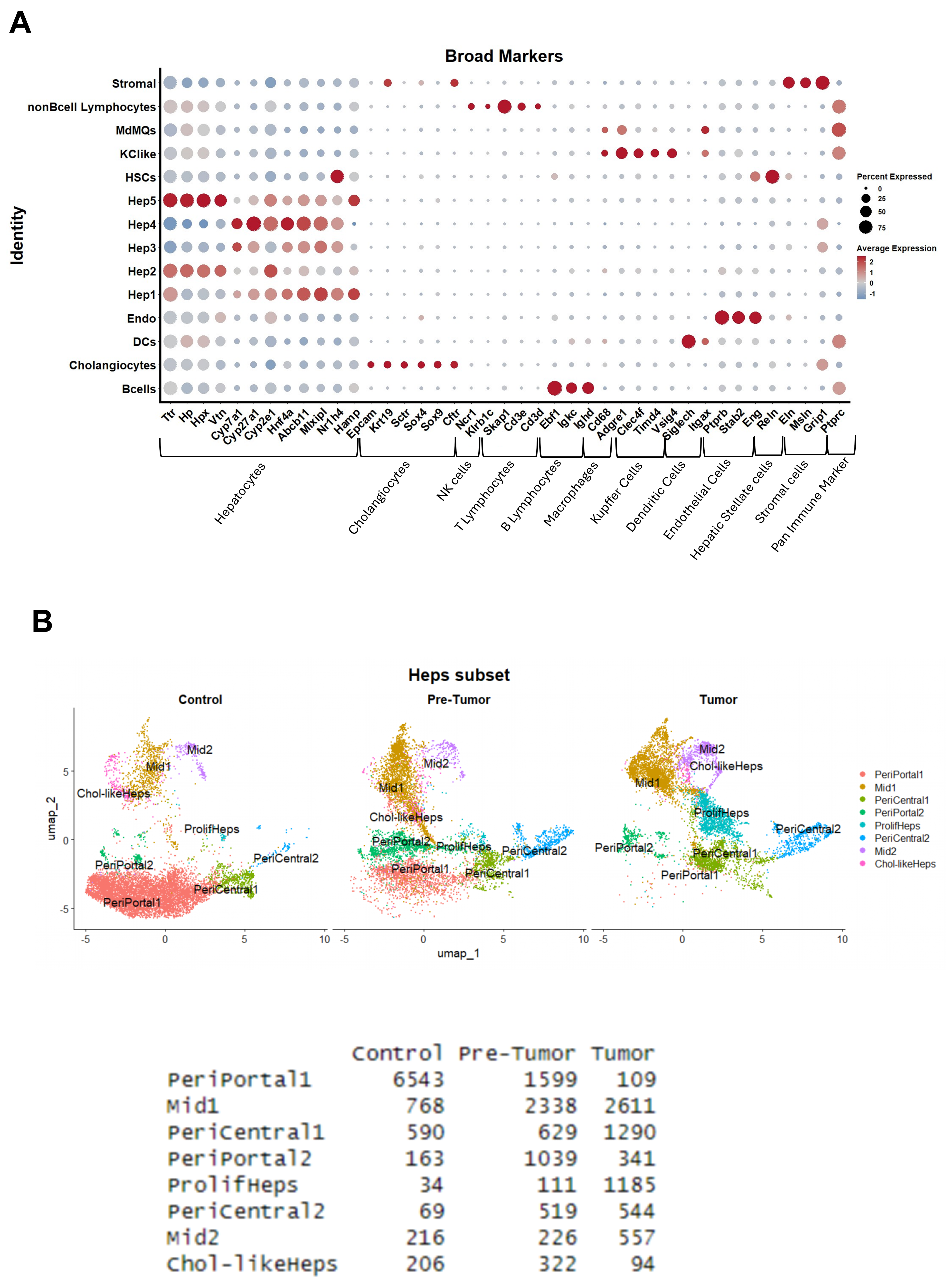

Supplement: Supplementary file 7 — Supplementary Material 7 [file 13578_2025_1362_MOESM7_ESM.tiff]

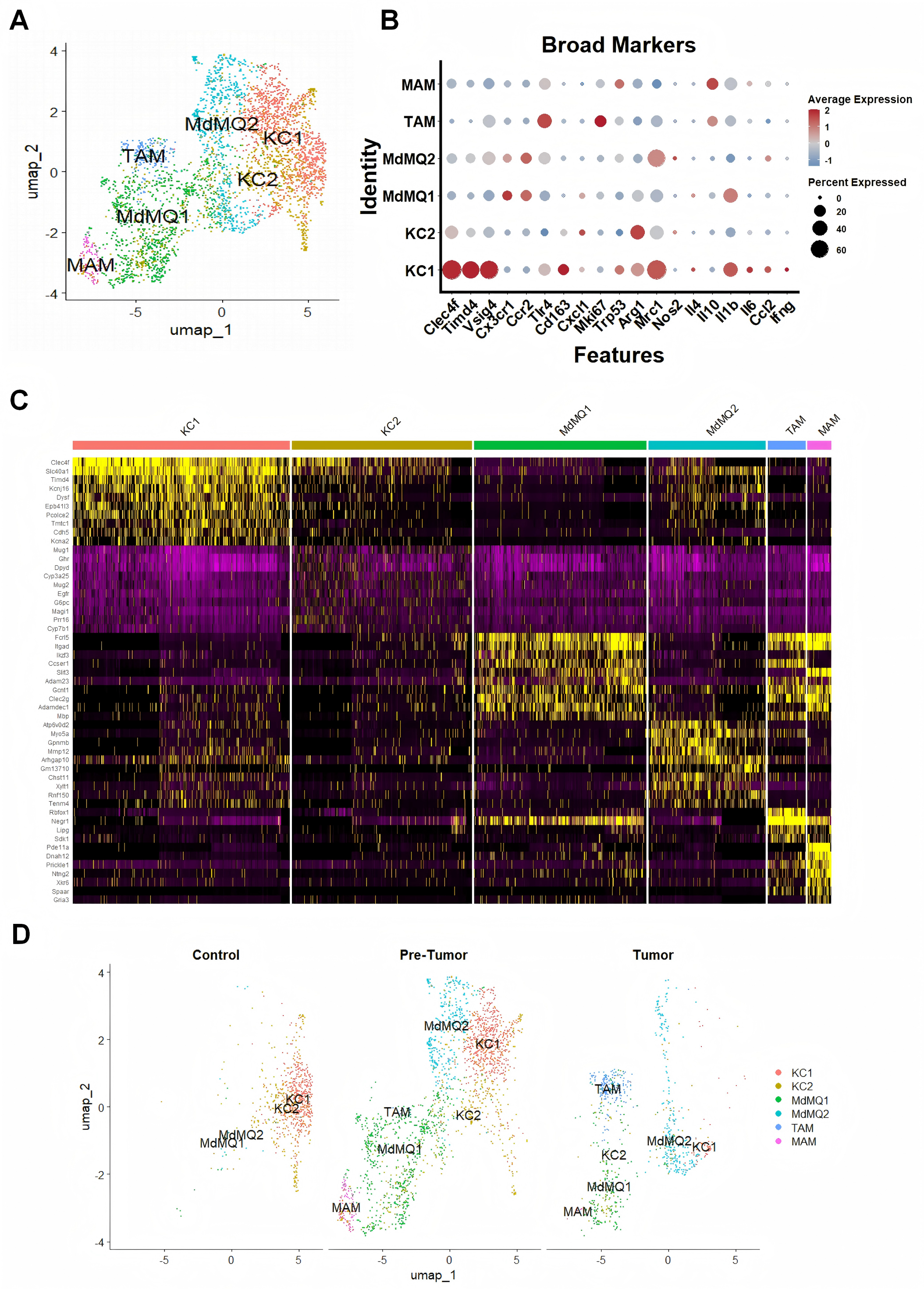

Supplement: Supplementary file 8 — Supplementary Material 8 [file 13578_2025_1362_MOESM8_ESM.tiff]

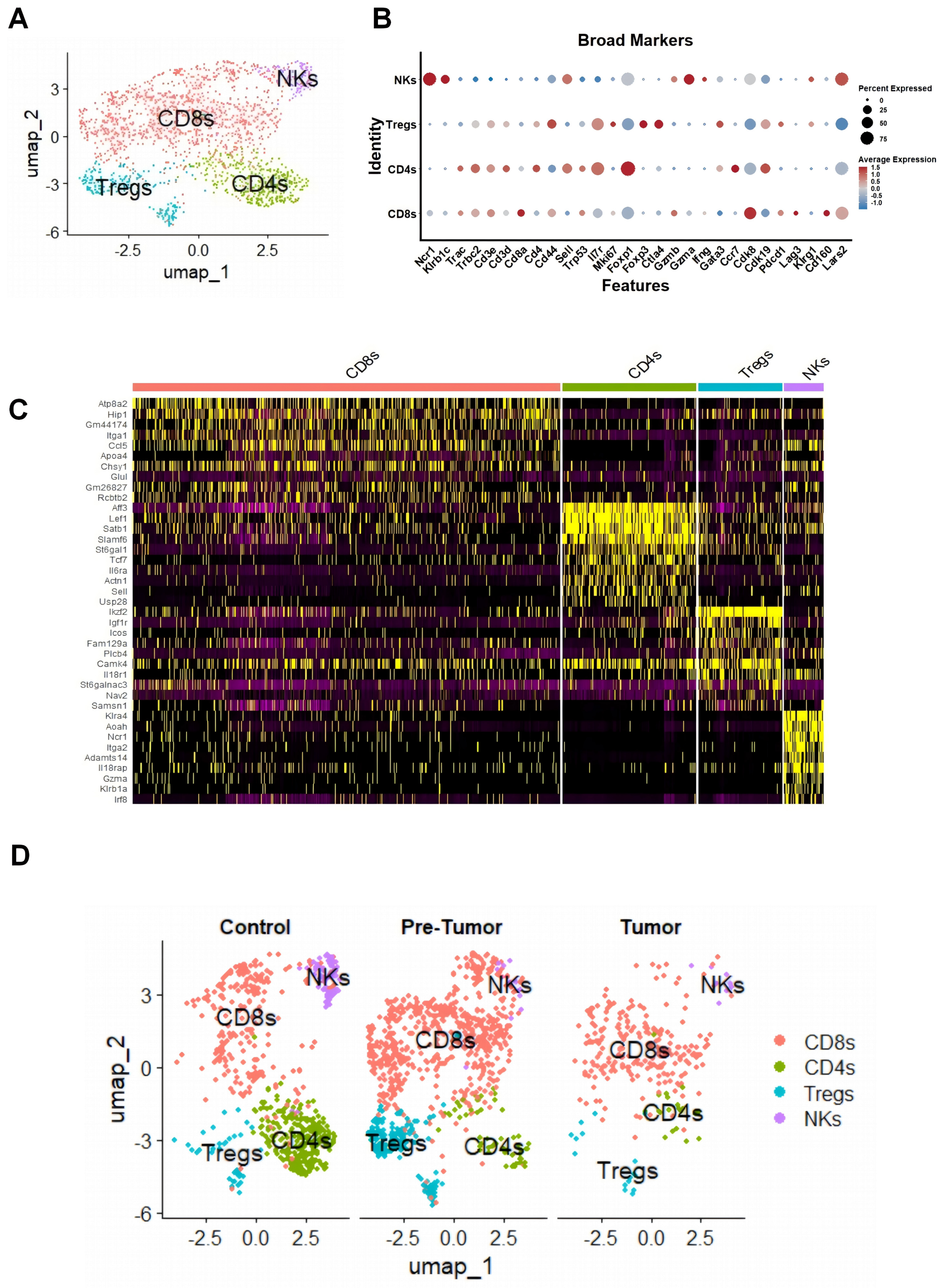

Supplement: Supplementary file 9 — Supplementary Material 9 [file 13578_2025_1362_MOESM9_ESM.tiff]

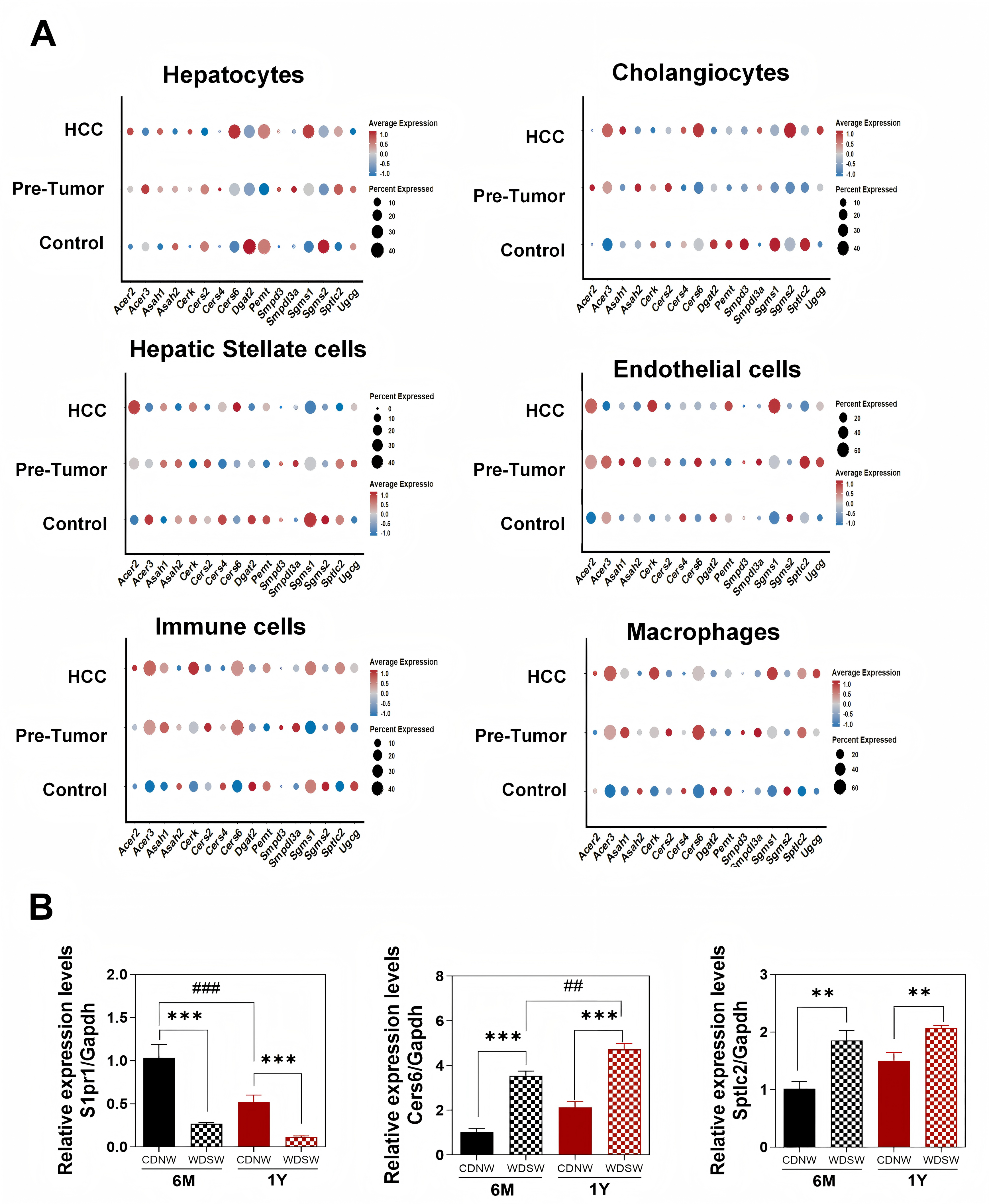

Supplement: Supplementary file 10 — Supplementary Material 10 [file 13578_2025_1362_MOESM10_ESM.tiff]

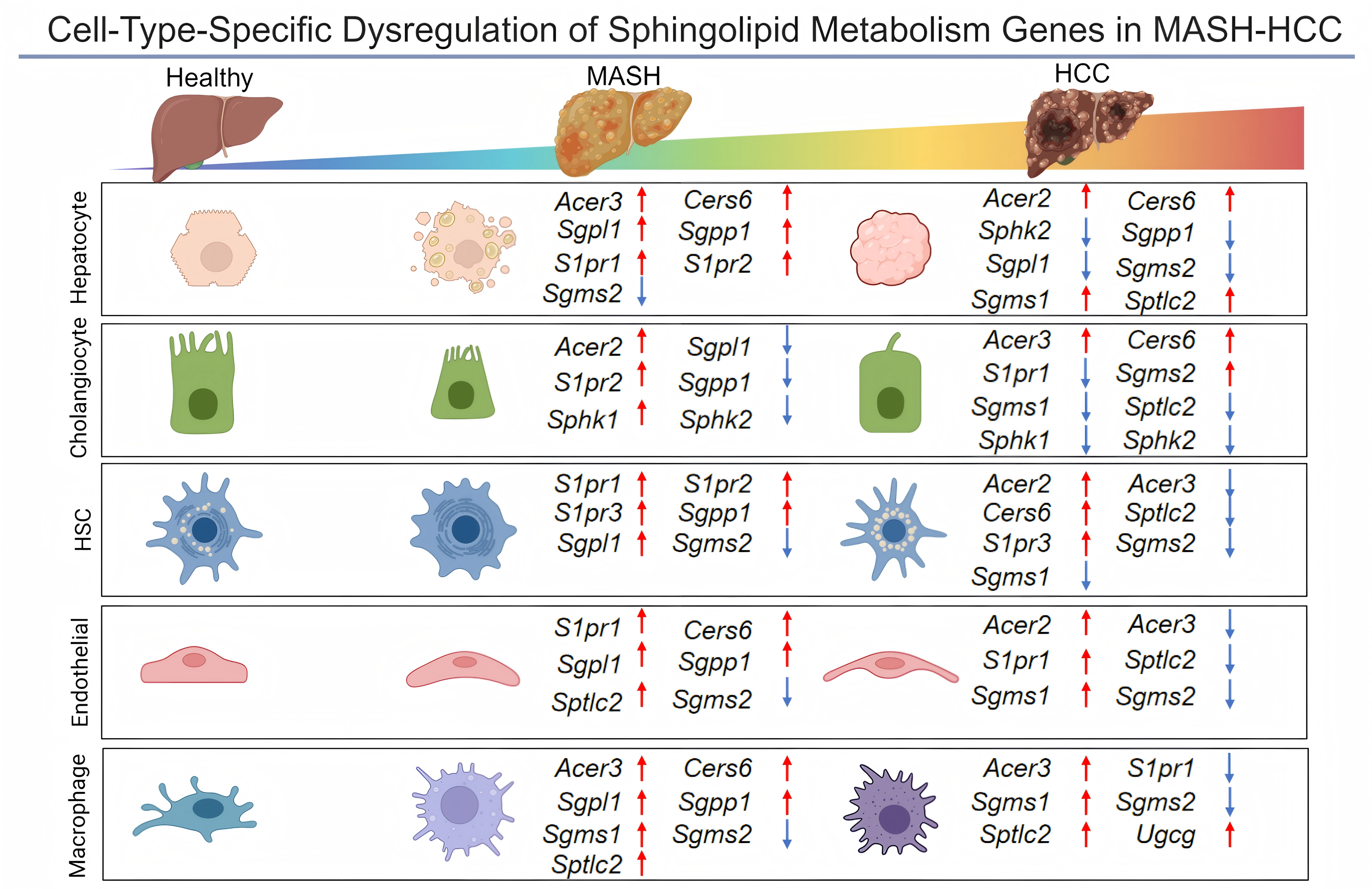

Supplement: Supplementary file 11 — Supplementary Material 11 [file 13578_2025_1362_MOESM11_ESM.tiff]
