# Supplementary material for: Transcriptomics, lipidomics, and single-nucleus RNA sequencing integration: exploring sphingolipids in MASH-HCC progression
Source: Cell Biosci. 2025 Mar 8;15:34. doi: 10.1186/s13578-025-01362-5 (PMC11890728; doi:10.1186/s13578-025-01362-5)
Supplement: Supplementary file 12 — Supplementary Material 12 [file 13578_2025_1362_MOESM12_ESM.docx]

**Supplementary Methods**

**Animal Experiments**

In this study, DIAMOND mouse models derived from a hybrid strain of C57BL/6J and 129S1/SvlmJ (ages 21 to 24 weeks, inclusive of both sexes) were utilized (1). The mice were divided into two dietary groups: one receiving a control diet with standard water (CDNW) and the other consuming a Western diet (supplied by Harlan, TD88137) paired with a sugar solution (23.1 g/L D-fructose and 18.9 g/L D-glucose) (WDSW). Both groups were allowed to feed freely over periods of 6 or 12 months. The animals were kept in a 12-hour light/dark cycle and in a temperature-controlled room with a constantly monitored 21 to 23℃ room temperature with continuous access to water. All the animal protocols were approved by the Institutional Animal Care and Use Committee of the Richmond VA Medical Center and Virginia Commonwealth University, and the experiments were conducted with the ethical standard for animal research. At the end of the experiments, mice were weighed and anesthetized by inhaled isoflurane before taking blood via cardiac puncture. The serum was immediately collected and frozen at −80℃ for later analysis. After the animals were sacrificed, the liver was collected for histological analyses and total RNA and protein extraction (2).

**Serum Biochemical Analysis**

The concentrations of alkaline phosphatase (ALP), aspartate aminotransferase (AST), alanine aminotransferase (ALT), and total cholesterol (CHOL) in the serum were measured using the Alfa Wassermann Vet ACE Axcel® System. Commercially provided assay kits from Alfa Wassermann Diagnostic Technologies, located in NJ, USA, were utilized in accordance with the manufacturer's guidelines (2, 3).

**Histological Staining**

Liver samples from mice were collected, fixed with 4% formaldehyde, embedded in paraffin, processed for hematoxylin and eosin (H&E) staining at the Mouse Model Core, Virginia Commonwealth University Massey Cancer Center in Richmond, VA, USA, and then scanned with a Vectra® Polaris Automated Quantitative Pathology Imaging System from Akoya Biosciences, Marlborough, MA, USA. Image capture was performed using the Phenochart software provided by Akoya Biosciences, MA, USA.

**RNA Sequencing (RNAseq) and Bioinformatic Analysis**

The total RNA was isolated from tissues using TRIzol Reagent (QIAGEN, Valencia, CA, USA). RNAseq with ribosomal RNA (rRNA) depletion was performed by Creative Biogene with the Illumina Hiseq® X (Shirley, NJ, USA). Cutadapt software was employed to eliminate reads with adapter contamination, low-quality bases, and ambiguous bases. Sequence integrity was confirmed using FastQC. The filtered sequences are mapped to the reference genome by Hisat2. FeatureCounts were used in the quantification and annotation of protein-coding genes. The analysis of differentially expressed genes (DEGs) included functional enrichment assessments, such as Gene Ontology (GO) analysis covering biological processes (BP), cellular components (CC), molecular functions (MF) and pathways in the Kyoto Encyclopedia of Genes and Genomes (KEGG) (3).

**Single-nucleus RNA-Sequencing data analysis**

A single nuclear RNA sequencing (snRNAseq) dataset was downloaded from GEO (GSE225381) and analyzed using the Seurat v5.0.3 package and followed the Seurat pipeline (4). Nuclei that had fewer than 200 genes, greater than 5,000 unique genes, and greater than 5% mitochondrial gene percentage were removed to eliminate empty droplets, doublets, ambient RNA contaminated droplets, or dying cells, leaving 39,017 nuclei for analysis (15,004 Control, 13,256 Pre-Tumor, and 10,757 HCC). Genes that were expressed in fewer than 3 nuclei were also removed from the analysis. SnRNAseq analysis then followed the Seurat SCTransform analysis methodology, regressing out mitochondrial gene percentage and utilizing Harmony integration.

Following principal component analysis (PCA), Harmony integration, uniform manifold approximation and projection (UMAP), and clustering, hemoglobin genes (Hba-a1, Hba-a2, and Hbb-bt) were used to identify ambient RNA contaminated events (5, 6). Their co-expression identified a contaminated cluster (each hemoglobin gene expressed in >75% of the cluster at high expression levels) accounting for 280 nuclei across all conditions (41 Control, 60 Pre-Tumor, and 179 HCC) ([arXiv:1802.03426](https://arxiv.org/abs/1802.03426)). This cluster was dropped due to ambient RNA contamination, leaving 38,737 nuclei for downstream analyses. Gene expression of classical markers was visualized using Seurat's DotPlot and VlnPlot for cell cluster identification. Heatmaps were generated using the DoHeatmap function of Seurat and differential gene expression and marker identification were performed using the FindMarkers and FindAllMarkers Seurat functions (7).

**Total RNA Isolation and Quantitative RT-PCR**

Total RNA was isolated using TRIzol Reagent (QIAGEN, Valencia, CA, USA) and reverse transcribed into cDNA using the High-Capacity cDNA Archive Kit (Applied Biosystems, Foster City, CA). The mRNA expression levels of specific genes were quantified using real-time RT-PCR as described previously (2). Specific primer sequences used for real-time RT PCR will be provided upon request.

**Human Liver Samples**

Frozen liver tissues from healthy individuals, MASH patients, and MASH-HCC patients were sourced from the Liver Tissue Cell Distribution System located in Minneapolis, MN. This system is supported by funding from the National Institutes of Health under contract number HSN276201200017C.

**Human TCGA Data Analysis**

RNA-seq data and clinical details for 374 HCC patients were obtained from The Cancer Genome Atlas (TCGA) through the TCGA-LIHC cohort-FPKM, accessible at <http://portal.gdc.cancer.gov/>. This cohort comprised individuals with primary tumors who underwent tumor resection. Additionally, there was an adjacent non-tumoral (NT) tissue group (n=50) for comparison with tumor tissue, aiming to calculate the individual fold changes (FCs) of corresponding genes.

**Analysis of Differentially Expressed Sphingolipid Metabolism-Related Genes**

To unravel the complex gene expression patterns and alterations within the liver tissues, we employed the NanoString nCounter® Metabolic Gene Expression panel with a customized sphingolipid metabolism panel, a comprehensive platform that covers the intricate pathways of sphingolipid metabolism. The DEGs were identified among the experimental groups using the Rosalind software. SMGs were selected based on their association with specific pathways identified through Gene Set Enrichment Analysis (GSEA), accessible at https://www.gsea-msigdb.org/. To determine the SMGs that affect the development and progression of HCC within the TCGA-LIHC cohort, DEGs between tumor and normal tissues were examined using the "edgeR" package. DEGs that exhibited an adjusted p-value < 0.05 and FC > 1.5 were used as screening criteria. Then, the DE-SMGs were identified in the DEGs.

**Development and Reliability Evaluation of Sphingolipid Metabolism-Related Prognosis-Related Signature in HCC**

The longest follow-up time or the duration of being known alive for patients was utilized for survival analysis. Gene expression levels were divided into quartiles, and the 1st and 4th quartiles representing low and high expression groups, respectively. The overall survival of these high and low-expression groups was compared using univariate Cox analysis implemented in R. The key genes associated with survival were identified based on adjusted p-values <0.05. The hazard ratio (HR) and 95% confidence intervals (CIs) were calculated.

The identified genes were then selected for multivariate Cox analysis. A risk score signature composed of three genes was established using data from TCGA-LIHC cohort. Based on the median of the risk score, patients were classified into high-risk and low-risk groups. The Kaplan–Meier survival curve was used to assess the predictive power of the prognosis model.

**Figure Legends**

**Figure S1. Total RNA Transcriptomic Profiling of Differentially Expressed Genes in the Liver of DIAMOND Mice during MASH-HCC Disease Progression.** Liver RNA samples from each experimental group underwent total RNA sequencing (n=3). DEGs were identified between different mice groups using FC and p-values (FC ≥1.5 and p-value <0.05). Hierarchical clustering heatmaps (left) display DEGs. Volcano plots (right) show gene expression differences. (A) Comparison between WDSW-6M (MASH) and CDNW-6M (Control). **(B)** Comparison between WDSW-1Y (HCC) and CDNW-1Y (Control). **(C)** Comparison between WDSW-1Y (HCC) and WDSW-6M (MASH). DIMOND: Diet-Induced MASLD Animal Model; MASH: Metabolic Dysfunction-Associated Steatohepatitis; HCC: Hepatocellular Carcinoma; DEG: Differentially Expressed Gene; FC: Fold Change; WDSW: Western Diet and Sugar Water; CDNW: Control Diet and Normal Water.

**Figure S2. Gene Ontology Analysis of Differentially Expressed Genes in DIAMOND Mice During MASH-HCC Disease Progression. (A)** Comparison of MASH vs Control. **(B)** Comparison of HCC vs Control.

**Figure S3. KEGG Pathway Analysis of Differentially Expressed Genes in DIAMOND Mice During MASH-HCC Disease Progression.** Comparison of MASH vs Control. **(B)** Comparison of HCC vs Control. KEGG: Kyoto Encyclopedia of Genes and Genomes.

**Figure S4. NanoString Profiling and Functional Analysis of Differentially Expressed Genes involved in hepatic and sphingolipid metabolism in the Liver of DIAMOND MASH Mice during MASH-HCC Disease Progression.** Liver RNA samples from each experimental group (n=3) were analyzed using NanoString nCounter metabolic mRNA panel with a customized sphingolipid metabolic panel. DEGs were identified in different groups using FC and p-values (FC ≥1.5 and p-value <0.05). **(A)** Volcano plots show gene expression differences between WDSW-6M and CDNW-6M (left) and WDSW-1Y and CDNW-1Y (right). **(B)** Gene Ontology Biological Process (GO-BP) analysis, **(C)** Gene Ontology Cellular Component (GO-CC) analysis, **(D)** Gene Ontology Molecular Function (GO-MF) analysis, and **(E)** KEGG analysis of DEGs between WDSW-6M and CDNW-6M (left), and WDSW-1Y and CDNW-1Y (right).

**Figure S5. Identification of Key Sphingolipid Metabolism-Related Genes in the Liver of DIAMOND Mice during MASH-HCC Disease Progression. (A)** Venn diagrams illustrate the upregulated key sphingolipid metabolism-related genes in MASH and HCC groups, including overlapping genes. **(B)** Venn diagrams illustrate the downregulated key sphingolipid metabolism-related genes in MASH and HCC groups, including overlapping genes. Key genes: S1pr1 (sphingosine-1-phosphate receptor 1), Sphk2 (sphingosine kinase 2), Sgpl1 (sphingosine-1-phosphate lyase 1), Smpd3 (sphingomyelin phosphodiesterase 3), Asah2 (neutral ceramidase), Cers6 (ceramide synthase 6), Acer2 (alkaline ceramidase 2), Elovl6 (elongation of very long chain fatty acid 6), Sgpp1 (sphingosine-1-phosphate phosphatase 1), Ugcg (glucosylceramide synthase), Cerk (ceramide kinase), Asah1 (acid ceramidase**).**

**Figure S6. Uniform Manifold Approximation and Projection (UMAP) Analysis of snRNAseq data Illustrating Cell Clustering Under Different Conditions**. (A) Control, (B) Pre-Tumor, and (C) Hepatocellular Carcinoma (HCC). Key cell types are labeled as follows: DCs (Dendritic Cells), Endo (Endothelial Cells), Hep (Hepatocytes), KClike (Kupffer Cell-like), and MdMQs (Monocyte-derived Macrophages).

**Figure S7**. A. Dot plot showing the percentage of cells expressing specific markers and their relative expression level for each cell cluster. DCs (Dendritic Cells), Endo (Endothelial Cells), Hep (Hepatocytes), KClike (Kupffer Cell-like), and MdMQs (Monocyte-derived Macrophages). B. UMAP of Hepatocyte sub-clustering segregated by condition with a data table showing hepatocyte subtyping counts for each condition.

**Figure S8**. A. UMAP showing sub-clustering of the monocyte-derived macrophages (MdMQs), Kupffer cells (KC), Tumor-associated macrophages (TAM) and MASH-associated macrophages (MAM). B. Dot plot showing the percentage of cells expressing specific markers and their relative expression levels for macrophage sub-clustering. C. Heatmap of the top genes associated with each macrophage subtype, with yellow indicating increased expression and purple showing decreased expression. D. UMAP from A segmented by different conditions.

**Figure S9**. A. UMAP showing sub-clustering of the non-B cell lymphocytes. B. Dot plot showing the percentage of cells expressing specific markers and their relative expression levels for non-B cell lymphocytes sub-clustering. C. Heatmap of the top genes associated with each subtype of the non-B cell lymphocytes subcluster, with yellow indicating increased expression and purple indicating decreased expression). D. UMAPs from A segmented by different conditions. (Control, Pre-Tumor, or HCC).

**Figure S10**. A. Dot plots for different cell types, showing the percentage of cells expressing key genes involved in ceramide metabolism and the relative expression level of each gene under different conditions (Control, Pre-Tumor, or HCC). In hepatocytes, Acer2^###^, Asah1^#^, Cerk^###^, Cers6^###^,Sgms1^###^, and Sptlc2^###^ were significantly upregulated in HCC vs Control, while Acer3^###^, Cers2^###^, and Dgat2^###^ were significantly downregulated. In cholangiocytes, Dgat2* and Smpd3** expression were significantly reduced in HCC vs Control, while Sgms2* and Ugcg** were significantly increased in HCC vs control. In HSCs, Acer2^###^ and Cers6*** were significantly upregulated in HCC vs Control, while Acer3*, Cers2*, Dgat2**, Smpd3*, Sgms1*** and Sgms2*** were significantly downregulated in HCC vs Control. In endothelial cells, Acer2^###^, Cerk^###^, Cers6*, Pemt*, Sgms1^###^, and Ugcg^###^ were significantly upregulated in HCC vs control, while Acer3^###^, Dgat2^###^, Sgms2^###^, and Sptlc2* were significantly downregulate in HCC vs control. In immune cells, Acer3^###^, Asah1^###^, Cerk^###^, Cers6^###^, and Sptlc2*** were significantly upregulated in HCC vs Control, while Cers4^#^, Dgat2***, and Sgms2*** were significantly reduced in HCC vs Control. In macrophages, Acer3^###^, Cerk^###^, Cers6***, Sgms1***, Sptlc2***, and Ugcg^###^ were significantly upregulated in HCC vs control, while Asah2***, Cers4^#^, and Sgms2*** were significantly downregulated in HCC vs Control. Statistical significance: *p<0.05, **p<0.01, ***p<0.001, ^#^adj p < 0.05, ^##^adj p < 0.01, ^###^adj p < 0.001. B. Relative mRNA expression levels of S1pr1, Cers6 and Sptlc2 in MASH and HCC mouse models. The mRNA levels were determined by qRT–PCR and normalized with Gapdh as an internal control. Data are expressed as the mean *±* SEM. Statistical significance relative to CDNW: ***p* < 0.01, ****p* < 0.001 (n=6-9).

**Figure S11**. **Cell-Type-Specific Dysregulation of Sphingolipid Metabolism Genes in MASH-HCC.** The image describes the dysregulation of the key sphingolipid genes across different cell types (hepatocyte, cholangiocyte, HSC, endothelial, and macrophage) under different conditions (Control, Pre-Tumor, or HCC). The arrows indicate the direction of change for each gene, with red arrows pointing up (indicating increased expression) and blue arrows pointing down (indicating decreased expression). The various genes involved are listed for each cell type, showing how they are affected in different stages of the disease.

**References**

1. Asgharpour A, Cazanave SC, Pacana T, Seneshaw M, Vincent R, Banini BA, Kumar DP, et al. A diet-induced animal model of non-alcoholic fatty liver disease and hepatocellular cancer. Journal of Hepatology 2016;65:579-588.

2. Wang Y, Tai Y-L, Zhao D, Zhang Y, Yan J, Kakiyama G, Wang X, et al. Berberine Prevents Disease Progression of Nonalcoholic Steatohepatitis through Modulating Multiple Pathways. Cells 2021;10.

3. Wang Y, Tai YL, Way G, Zeng J, Zhao D, Su L, Jiang X, et al. RNA binding protein HuR protects against NAFLD by suppressing long noncoding RNA H19 expression. Cell Biosci 2022;12:172.

4. Hao Y, Stuart T, Kowalski MH, Choudhary S, Hoffman P, Hartman A, Srivastava A, et al. Dictionary learning for integrative, multimodal and scalable single-cell analysis. Nat Biotechnol 2024;42:293-304.

5. Korsunsky I, Millard N, Fan J, Slowikowski K, Zhang F, Wei K, Baglaenko Y, et al. Fast, sensitive and accurate integration of single-cell data with Harmony. Nat Methods 2019;16:1289-1296.

6. Tan HS, Wang K, McBeth R. Exploring UMAP in hybrid models of entropy-based and representativeness sampling for active learning in biomedical segmentation. Comput Biol Med 2024;176:108605.

7. Jiang X, Xu Y, Fagan A, Patel B, Zhou H, Bajaj JS. Single nuclear RNA sequencing of terminal ileum in patients with cirrhosis demonstrates multi-faceted alterations in the intestinal barrier. Cell Biosci 2024;14:25.
